# DeSUMOylating isopeptidase 1 participates in the faithful chromosome segregation and vincristine sensitivity

**DOI:** 10.1096/fj.202401560RR

**Published:** 2024-12-19

**Authors:** Yuki Ikeda, Ryuzaburo Yuki, Youhei Saito, Yuji Nakayama

**Affiliations:** ^1^ Laboratory of Biochemistry & Molecular Biology Kyoto Pharmaceutical University Kyoto Japan

**Keywords:** Aurora B, DESI1, deSUMOylase, FoxM1, spindle assembly checkpoint, SUMO, vincristine

## Abstract

SUMOylation, the modification of proteins with a small ubiquitin‐like modifier (SUMO), is known to regulate various cellular events, including cell division. This process is dynamic, with its status depending on the balance between SUMOylation and deSUMOylation. While the regulation of cell division by sentrin‐specific protease (SENP) family proteins through deSUMOylation has been investigated, the role of another deSUMOylase, deSUMOylating isopeptidase 1 (DESI1), remains unknown. In this study, we explored DESI1's role in cell division. Knockdown of DESI1 accelerated cell division progression, leading to a significant increase in abnormal chromosome segregation. These phenotypes were rescued by re‐expression of wild‐type DESI1, but not catalytically inactive DESI1. DESI1 knockdown reduced the mitotic arrest caused by nocodazole, suggesting DESI1's involvement in the spindle assembly checkpoint (SAC). Localization of Aurora B, a key SAC regulator, at the metaphase chromosomes was reduced due to decreased Aurora B expression upon DESI1 knockdown. Consistently, DESI1 knockdown reduced transcription of FoxM1 target genes, such as Aurora B, cyclin B1, and CENP‐F. The TCGA database showed that both decreased and increased DESI1 expression levels are associated with poor prognosis in patients with certain cancer types. Importantly, we found that DESI1 knockdown reduced sensitivity to vincristine by inducing mitotic slippage. These results suggest that DESI1 is required for faithful chromosome segregation via regulating FoxM1 transcriptional activity and thereby SAC activity in an isopeptidase activity‐dependent manner. Our findings identified DESI1 as a novel regulator of cell division and a factor affecting cancer chemotherapy.

## Graphical abstract

DeSUMOylating isopeptidase 1 (DESI1), a member of the second class of SUMO protease, maintains the expression level of Aurora B to promote the spindle assembly checkpoint. DESI1 is required for the activity of FoxM1 in the transcription of mitotic regulators including Aurora B. Lower levels of DESI1 in cancer cells are associated with poorer prognosis of patients with endometrioid cancer and a decrease in sensitivity against microtubule‐targeting agents. Our findings suggest that DESI1 may play a crucial role in the maintenance of chromosomal integrity.

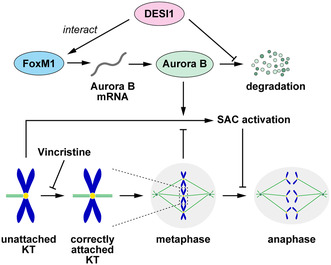

**Abbreviations**
3′‐UTR: 3′‐untranslated region
APC/C: anaphase‐promoting complex/cyclosome
BubR1: budding uninhibited by benzimidazole‐related 1
Cdc20: cell division cycle 20
Cdh1: Cdc20 homolog 1
CDK1: cyclin‐dependent kinase 1
CENP: centromere‐associated protein
DESI1: deSUMOylating isopeptidase 1
Dox: doxycycline
FBS: fetal bovine serum
FoxM1: forkhead box M1
HA: hemagglutinin
HRP: horseradish peroxidase
INCENP: inner centromere protein
JNK: Jun N‐terminal kinase
Mad2: mitotic arrest deficient 2
MCC: mitotic checkpoint complex
MELT: Met‐Glu‐Leu‐Thr
MPS1: monopolar spindle 1
PIAS: protein inhibitor of activated STAT
PMSF: phenylmethylsulfonyl fluoride
RanBP2: Ran‐binding protein 2
RNF4: ring finger protein 4
SAC: spindle assembly checkpoint
SAE1: SUMO‐activating enzyme subunit 1
SAE‐2: SUMO‐activating enzyme subunit 2
SENP: sentrin‐specific protease
si: small interfering
STLC: S‐trityl‐L‐cysteine
SUMO: small ubiquitin‐like modifier
TopoII: topoisomerase 2
VCR: vincristine
WT: wild‐type

## 1 INTRODUCTION

Cell division, the most dynamic process among cellular events, involves the equal separation of duplicated chromosomes into two daughter cells. Upon mitotic entry, condensed chromosomes are captured by microtubules emanating from centrosomes. Chromosomes are aligned at the mitotic spindle equator, powered by the microtubule‐associated motor protein, such as CENP‐E,^1^, ^2^ and by microtubule dynamics such as polymerization and depolymerization at kinetochores.^3^ The Knl1/Mis12 complex/Ndc80 complex network is essential for kinetochore‐microtubule interactions.^4^ Until all sister kinetochores are properly connected with spindle microtubules, the spindle assembly checkpoint (SAC) halts the onset of anaphase.^5^ The key SAC regulators include Aurora B and MPS1 kinases. Aurora B helps SAC signaling by facilitating MPS1 recruitment to unattached kinetochores.^6^, ^7^ MPS1 phosphorylates the MELT repeats, Met‐Glu‐Leu‐Thr motifs in Knl1, thereby establishing docking sites for the assembly of the SAC effector, the mitotic checkpoint complex (MCC) consisting of Mad2, Cdc20, BubR1, and Bub3.^5^, ^8^, ^9^ The MCC inhibits APC/C‐Cdc20 and thereby prevents polyubiquitinated protein degradation, including cyclin B1 and securin, thereby delaying the anaphase onset. A defect in this checkpoint, including Aurora B inactivation, causes a premature anaphase onset with errors in chromosome segregation, known as mitotic slippage.^10^, ^11^ When cancer cells undergo mitotic slippage in the presence of the microtubule‐targeting agents, such as vincristine and paclitaxel, cancer cells avoid subsequent cell death. This leads to the induction of chromosomal instability through polyploidy, which is linked to cancer malignant progression.^11^, ^12^, ^13^ Once the checkpoint is satisfied by proper kinetochore–microtubule interactions, APC/C triggers cyclin B1 degradation. Topoisomerase II‐decatenated sister chromatids are segregated toward opposite poles,^14^ and the nuclear envelope reassembles around the decondensed chromosomes. Finally, the cytoplasm is split into two daughter cells during cytokinesis.

Post‐translational protein modifications, including phosphorylation and ubiquitination, play critical roles in regulating cell division.^15^ SUMOylation, a process that modifies proteins with Small Ubiquitin‐Like Modifier (SUMO) at lysine residues, regulates a variety of cellular events, including gene expression, DNA repair, nucleocytoplasmic trafficking, cell cycle progression, and proteolysis.^16^ Alongside phosphorylation and ubiquitination, SUMOylation also regulates chromosome segregation.^17^ SUMOylation is achieved through an enzymatic cascade involving a heterodimeric E1‐activating enzyme (SAE1‐SAE2), an E2‐conjugating enzyme (UBC9), and E3 ligases, which include protein inhibitors of activated STAT (PIAS) family members and Ran‐binding protein 2 (RanBP2).^17^ The SUMOylation of key regulators of cell division, including Aurora B,^18^, ^19^ FoxM1,^20^, ^21^ topoisomerase II (Topo II),^22^ and CENP‐E,^23^ has been reported, and their SUMOylation levels are critical for their functions. SUMOylation is dynamic, and its status depends on the balance between SUMOylation and deSUMOylation.^24^ deSUMOylation is a fundamental regulator of cell division, as is SUMOylation.^25^ Consistent with the critical roles of SUMOylation and deSUMOylation in cell cycle progression, deregulation of SUMO modification status is associated with cancer.^17^ Overexpression of proteins regulating SUMO modification, including SUMO1, SAE2, UBC9, PIASs, RanBP2, and sentrin‐specific proteases (SENPs), has been observed in various cancer types.^17^ SENP family proteins are cysteine proteases and catalyze the deconjugation of SUMO from SUMOylated proteins.^24^

The deSUMOylating isopeptidase 1 (DESI1) has been identified as a secondary class of SUMO protease.^26^ In addition to the C‐terminal catalytic domain, SENPs possess an N‐terminal non‐catalytic region that directs their subcellular localization. However, unlike SENPs, DESI1 and DESI2 lack this corresponding region. Contrast with SENPs, which localize to the nucleus, nucleolus, and nuclear pore in interphase and to the kinetochore in M phase,^24^ DESIs are distributed throughout the cytoplasm and nucleus (DESI1) or are restricted to the cytoplasm (DESI2).^26^ The substrate specificity of DESI1 differs from that of SENPs. DESI1 exhibits endopeptidase activity toward precursor forms of SUMO‐1 and SUMO‐2, albeit with extremely low activity.^27^ In certain cancer types, mutations and amplifications of the DESI1 gene have been identified in the c‐BioPortal database (Figure S1) (https://www.cbioportal.org), suggesting that, like SENPs, DESI1 could be involved in the regulation of cell division and its deregulation could be associated with oncogenesis via the induction of chromosomal instability. However, whether DESI1 participates in oncogenesis remains unknown. In the current study, we demonstrate that DESI1 knockdown causes acceleration of M‐phase progression with abnormal chromosome segregation, including chromosome bridges and lagging chromosomes in anaphase cells. This was accompanied by reduced localization of Aurora B on the chromosomes due to suppression of FoxM1 transcriptional activity. The results of this study indicate that DESI1 is a novel regulator of cell division, catalyzing deSUMOylation, and that its deregulation may be associated with cancer by causing failure in cell division.

## 2 MATERIALS AND METHODS

### 2.1 Cells

Human cervical cancer HeLa S3, endometrioid cancer HEC‐1‐A (Japanese Collection of Research Bioresources, Osaka, Japan), and Lenti‐X 293T cells (Clontech Laboratories, Mountain View, CA, USA) were cultured in Dulbecco's modified Eagle medium, supplemented with 5% (HeLa S3, Lenti‐X 293T) or 10% (HEC‐1‐A) fetal bovine serum (FBS), 20 mM HEPES‐NaOH (pH 7.4), and 2 mM of L (+)‐glutamine at 37°C in a 5% CO_2_ environment. Lenti‐X 293T cells were utilized for virus production.

### 2.2 siRNA

HeLa S3 and HEC‐1‐A cells were transfected with small interfering RNA (siRNA) using Lipofectamine 2000 reagent (Thermo Fisher Scientific, Waltham, MA, USA). For DESI1 knockdown, 10 or 20 pmol of siRNA/well of 24‐well plate and 5 pmol of siRNA/well of 96‐well plate were used. For SENP1 and FoxM1 knockdown, 10 and 125 pmol of siRNA/well of 24‐well plate, respectively, were used. siDESI1 #1 (5′‐CUGACUGGGCGGAAGAUUC‐3′), #2 (5′‐GAGGUGAGGCCUACAACCU‐3′), siSENP1 (5′‐GAAACAGCCGAAGUCUUUA‐3′), and siFoxM1 (5′‐CAACUCUUCUCCCUCAGAU‐3′) were procured from MilliporeSigma (Burlington, MA, USA). siDESI1 #3 (5′‐GAUUUCUAUUUUAUAAUUU‐3′), targeted to the 3′‐UTR, was synthesized by MilliporeSigma. MISSION siRNA Universal Negative Control #1 (SIC‐001, MilliporeSigma) was used as control siRNA.

### 2.3 Plasmids

To construct the pENTR4‐no‐ccDB‐FLAG‐DESI1 plasmid, a KpnI–NotI fragment of the pCMV6‐Entry DESI1‐Myc‐DDK‐tagged (RC210892; OriGene Technologies, Rockville, MD, USA) was ligated into the KpnI and NotI sites of the pENTR4‐no‐ccDB (686‐1) [a gift from Eric Campeau & Paul Kaufman (Addgene plasmid #17424; http://n2t.net/addgene:17424; RRID:Addgene_17424)^28^], generating pENTR4‐no‐ccDB‐DESI1 (w/o stop). The pENTR4‐no‐ccDB‐DESI1 (w/o stop) was subjected to inverse PCR using primers (5′‐GCTAGGCCGCACTCGAGATATCTAGACCC‐3′ and 5′‐TCTGGCCGTTGGGTCTGCCCA‐3′) to introduce a stop codon (pENTR4‐no‐ccDB‐DESI1), and then inverse PCR using primers (5′‐GACGATAAGGCAGGTATGGAGCCGCCGAATCTCTATCCG‐3′ and 5′‐GTCATCCTTGTAATCCATGGTGGAGCCTGCTTTTTTGTAC‐3′) was performed. The amplified fragment was phosphorylated by T4 polynucleotide kinase (Toyobo, Cat #2021S, Osaka, Japan) and circularized by Ligation mix (#6023; Takara Bio, Shiga, Japan), generating pENTR4‐no‐ccDB‐FLAG‐DESI1. For the construction of the catalytically inactive mutant (C108S), Cys108 of DESI1 was substituted with Ser by inverse PCR using primers (5′‐CTAACACCTTCAGCAACGAAGTGGCACAGTTCCTGACTG‐3′ and 5′‐AATTGTGTTCAAAGAGGTTGTAGGCCTCACCTCGGAACAGGG‐3′) using the pENTR4‐no‐ccDB‐FLAG‐DESI1 as a template.

To construct the pENTR4‐no‐ccDB‐Strep‐HA‐DESI1 plasmid, first, the NcoI–XbaI fragment of the pcDNA3.1‐Strep‐HA‐SUMO2 [a gift from Ying Liu (Addgene plasmid #66867; http://n2t.net/addgene:66867; RRID:Addgene_66867)^29^] was ligated into the corresponding sites of the pENTR4‐no‐ccDB, generating the pENTR4‐no‐ccDB‐Strep‐HA‐SUMO2 plasmid. Then, SUMO2 was removed by inverse PCR with primers (5′‐AAGCTTAGATCTGCGGCCGCTCGAGTCTAGACCCAGCTTTC‐3′ and 5′‐GAGGATCCCTGCAGCGGCGTAATCGGGGACGTCATAAGGG‐3′), generating the pENTR4‐no‐ccDB‐Strep‐HA. Finally, a DESI1‐coding fragment with BamHI‐XhoI sites, amplified with primers (5′‐TTTGGATCCTCATGGAGCCGCCGAATCTCTATCCGG‐3′ and 5′‐TTTCTCGAGCTAGCTCTGGCCGTTGGGTCTGCC‐3′) using the pENTR4‐no‐ccDB‐DESI1 as a template, was ligated to the BamHI‐XhoI sites of the pENTR4‐no‐ccDB‐Strep‐HA, generating the pENTR4‐no‐ccDB‐Strep‐HA‐DESI1.

A fragment coding FoxM1 with BamHI‐XhoI sites, amplified with primers (5′‐AAAGGATCCTCAAAACTAGCCCCCGTCGGCCA‐3′ and 5′‐AAACTCGAGCTACTGTAGCTCAGGAATAAACTGGGACC‐3′) using the pCW57.1‐FLAG‐FoxM1c [a gift from Adam Karpf (Addgene plasmid #68810; http://n2t.net/addgene:68810; RRID:Addgene_68810)^30^] as a template, was ligated to the BamHI‐XhoI sites of the pENTR4‐no‐ccDB‐Strep‐HA, generating the pENTR4‐no‐ccDB‐Strep‐HA‐FoxM1.

To construct pENTR4‐no‐ccDB‐FLAG‐SENP1, a SENP1 fragment harboring SalI–XbaI sites, amplified with primers (5′‐AAAGTCGACACCATGGACTACAAAGACGATGACGACAAGC‐3′ and 5′‐AAATCTAGATCACAAGAGTTTTCGGTGGAGGATCTCCC‐3′) using the Flag‐SENP1 [a gift from Edward Yeh (Addgene plasmid #17357; http://n2t.net/addgene:17357; RRID:Addgene_17357)^31^], was ligated into the SalI–XbaI sites of the pENTR4‐no‐ccDB.

The genes in the entry vectors were subcloned into the gateway destination vectors, pLIX402 [a gift from David Root (Addgene plasmid #41394; http://n2t.net/addgene:41394; RRID:Addgene_41394)], pLX301 [a gift from David Root (Addgene plasmid #25895; http://n2t.net/addgene:25895; RRID:Addgene_25895)^32^], and pLX303 [a gift from David Root (Addgene plasmid #25897; http://n2t.net/addgene:25897; RRID:Addgene_25897)^32^] lentiviral plasmids using the Gateway LR reaction as per the manufacturer's instructions (Thermo Fisher Scientific).

### 2.4 Establishment of clone cells

The constructed pLIX_402 vectors were used to generate cells capable of doxycycline (Dox)‐inducible expression of proteins, and the pLX_301 and pLX_303 vectors were used for stable expression in cells. For viral production, Lenti‐X 293T cells were placed in 3.5 cm dishes and co‐transfected with either 1.2 μg of pLIX_402 FLAG‐DESI1 (generated cells were referred as HeLa S3/FLAG‐DESI1), pLIX402_FLAG‐DESI1 C108S (HeLa S3/FLAG‐DESI1 C108S), pLIX_402 Strep‐HA‐DESI1 (HeLaS3/Strep‐HA‐DESI1), pLIX_402 Strep‐HA‐FoxM1, pLX_303 FLAG‐DESI1 (HeLa S3/Strep‐HA‐FoxM1 and HeLa S3/Strep‐HA‐FoxM1/FLAG‐DESI1), or pLIX_402 FLAG‐SENP1 (HeLa S3/FLAG‐SENP1), along with 0.8 μg each of pCAG‐HIVgp and pCMV‐VSV‐G‐RSV‐Rev (gifted by Dr. Hiroyuki Miyoshi, Rikagaku Kenkyusho Foundation, BioResource Center, Ibaraki, Japan) using the Lipofectamine 2000 reagent. After a period of 16 h, the cells were exposed to 10 mM forskolin (067‐02191, FUJIFILM Wako, Osaka, Japan) and further cultured for an additional 8 h. The medium containing the virus was filtered through a 0.45 μm filter and added to the HeLa S3 cells that had been pretreated with 80 μg/mL polybrene (MilliporeSigma). The infected HeLa S3 cells were then selected with 2 μg/mL puromycin (StressMarq Biosciences, Victoria, BC, Canada) or 5 μg/mL blasticidin S (KK‐400, Kaken Pharmaceutical Co. Ltd., Tokyo, Japan).

### 2.5 Antibodies

The primary antibodies used for immunoblotting (IB) or immunofluorescence (IF) included the following: rabbit polyclonal anti‐DESI1 (IB, 1:800; R71217, Atlas Antibodies, Bromma, Stockholm, Sweden) and anti‐cyclin B1 (IB, 1:1000; H‐433, sc‐752, Santa Cruz Biotechnology, Dallas, TX, USA) antibodies; rabbit monoclonal anti‐phospho‐Aurora A (Thr288) /Aurora B (Thr232) / Aurora C (Thr198) antibody (IB, 1:1000; #2914S, Cell Signaling Technology, Danvers, MA, USA); mouse monoclonal anti‐AIM1 (Aurora B) (IB, 1:1000; IF, 1:800; 611 082, BD Transduction Laboratories, Lexington, KY, USA), anti‐FLAG (IB, 1:1000; M2, F1804, MilliporeSigma), anti‐FoxM1 (IB, 1:1000; G‐5, sc‐376 471, Santa Cruz Biotechnology), anti‐γ‐tubulin (IF, 1:250; GTU‐88, T6557, MilliporeSigma), anti‐phospho‐histone H3 (Ser10, IB, 1:1000; #9701S, Cell Signaling Technology), and anti‐SENP1 (IB, 1:500; C‐12, sc‐271 360, Santa Cruz Biotechnology) antibodies; rat monoclonal anti‐α‐tubulin (IB, 1:4000; IF, 1:800; YOL1/34, MCA78G, Bio‐Rad, Hercules, CA, USA) and anti‐HA (IB, 1:1000; 3F10, 11 867 423 001, Roche, Basel, Switzerland) antibodies.

For immunofluorescence analysis, Alexa Fluor 488‐labeled donkey anti‐mouse (IF, 1:800; A21202) and donkey anti‐rat (IF, 1:800, A21208) antibodies, and Alexa Fluor 555‐labeled goat anti‐rat (IF, 1:800; A21434) and donkey anti‐rat (IF, 1:800; A78945) antibodies were purchased from Thermo Fisher Scientific. For Western blot analysis, horseradish peroxidase (HRP)‐conjugated anti‐mouse (IB, 1:8000; 715‐035‐151), anti‐rabbit (IB, 1:8000; 711‐035‐152), and anti‐rat (IB, 1:8000; 712‐035‐153) antibodies were procured from Jackson Immuno Research (West Grove, PA, USA).

### 2.6 Immunofluorescence staining

Immunofluorescence staining was conducted on coverslips. Cells were fixed with 4% formaldehyde in PBS (−) for 20 min at room temperature. For γ‐tubulin staining, cells were fixed with 100% MeOH for 5 min at −30°C. The fixed cells were permeabilized and blocked with PBS (−) containing 3% bovine serum albumin and 0.1% saponin for 30 min. The cells were then incubated with primary and secondary antibodies, diluted with PBS (−) containing 3% BSA and 0.1% saponin, for 1 h each at room temperature. DNA was stained with 0.1–1 μM Hoechst 33342 during the incubation with the secondary antibody.

Fluorescence images were captured using an IX‐83 fluorescence microscope (Olympus, Tokyo, Japan) equipped with a 40× 0.75 NA objective lens and a 60× 1.42 NA oil‐immersion objective lens (Olympus). The optical system included U‐FUNA (360‐370 nm excitation, 420–460 nm emission), U‐FBNA (470–495 nm excitation, 510–550 nm emission), and U‐FRFP (535–555 nm excitation, 570–625 nm emission) cubes for obtaining Hoechst 33342, Alexa Fluor 488, and Alexa Fluor 555 fluorescence, respectively. Composite images were edited using Image J (National Institutes of Health, Bethesda, MD, USA), Photoshop CC, and Illustrator CC software (Adobe, San Jose, CA, USA). The fluorescence intensities of the images were quantified using ImageJ (National Institutes of Health).

### 2.7 Western blot analysis

Cells were lysed in a sodium dodecyl sulfate (SDS)‐sample buffer containing protease and phosphatase inhibitors (2–20 μg/mL aprotinin, 0.8–8 μg/mL pepstatin A, 2–20 μg/mL leupeptin, 0.5–5 mM EGTA‐KOH, 1–4 mM PMSF, 20 mM β‐glycerophosphate, 50 mM NaF, 10 mM Na_3_VO_4_) and were then boiled for 3 min at 100°C. The lysates were separated by SDS‐PAGE and transferred onto polyvinylidene difluoride membranes (BSP0161, Pall Corporation, Port Washington, NY, USA). The membrane was blocked using Blocking One (03953‐95, Nacalai Tesque, Kyoto, Japan) and incubated with primary and secondary antibodies diluted in Tris‐buffered saline containing 0.1% Tween 20 and 5% Blocking One for 1 to 2 h at room temperature or overnight at 4°C. To inactivate HRP for sequential probing, 0.1% NaN_3_ was added to the primary antibodies. Chemiluminescence was detected with the image analyzer ChemiDoc XRSplus (Bio‐Rad) using Chemi Lumi One L (#07880, Nacalai Tesque), Clarity (170‐5061, Bio‐Rad) and Chemi Lumi One Ultra (11644‐40, Nacalai Tesque) as substrates. Protein expression levels were analyzed by measuring the signal intensity of the bands using ImageJ.

### 2.8 Analysis of M‐phase progression

M‐phase progression was analyzed as outlined in previous reports.^33^, ^34^ Briefly, HeLa S3 cells were seeded in a 24‐well plate and transfected with 10 pmol siRNA. After 28 h, cells were treated with the reversible CDK1 inhibitor RO‐3306 (S7747, Selleck Chemicals, Houston, TX, USA)^35^ at 6 μM for 20 h. To release from RO‐3306, cells were washed four or five times on a 37°C water bath with pre‐warmed PBS containing Ca^2+^ and Mg^2+^ [PBS (+)] and incubated with pre‐warmed fresh medium for 45, 60, or 75 min. Following fixation with 4% formaldehyde in PBS (−), the cells were stained for α‐tubulin and DNA. Based on the microtubule and chromosome morphologies, cells were classified into four groups: prophase/prometaphase (P/PM), metaphase (M), anaphase/telophase (A/T), and cytokinesis (Cyto). The percentage of each category was then calculated.

### 2.9 Time‐lapse imaging

For time‐lapse imaging of cell division, cells were seeded in a 24‐well plate and synchronized to M phase using RO‐3306 as described above. Immediately after removal of RO‐3306, 0.1 μM Hoechst 333342 was added to the culture medium. Time‐lapse images of bright‐field and Hoechst 33342 fluorescence were captured at 5 min intervals in a live‐cell chamber at 37°C in 5% CO_2_ using the Operetta imaging system (PerkinElmer, Waltham, MA, USA). The durations of each mitotic subphase, such as P/PM, M, and A/T, were subsequently determined. Alternatively, cells were seeded in a 96‐well plate, and without synchronization, time‐lapse images were captured in the presence of 50 ng/mL nocodazole (146‐08533, FUJIFILM Wako) and 0.1 μM Hoechst 33342 at 20‐min intervals for 24 h.

### 2.10 Real‐time PCR

For real‐time PCR analysis, cells were seeded in a 24‐well plate and treated with siRNA for 24 h. To synthesize cells to the G2 phase, they were incubated with 4 mM thymidine (T1895‐10G, MilliporeSigma) for 24 h at 37°C and then cultured for an additional 9 h without thymidine. To harvest M‐phase cells, cells were treated with siRNA for 22 h and 5 μM STLC (164739‐5G, MilliporeSigma) during the final 16 h. The cells were collected by mitotic shake‐off.

Cells were lysed with DNaseI treatment, and reverse transcription into cDNA was performed using the TaqMan Fast Advanced Cells‐to‐CT Kit (A35374, Thermo Fisher Scientific) according to the manufacturer's instructions. Quantitative PCR was performed using TaqMan probe‐based gene expression analysis and QuantStudio 1 (Thermo Fisher Scientific). TaqMan probe‐based primers purchased from Thermo Fisher Scientific were used, including Aurora B (AURKB, Hs00945858_g1), cyclin B1 (CCNB1, Hs01030099_m1), CENP‐F (CENP‐F, Hs01118845_m1), and GAPDH (GAPDH, Hs02786624_g1). GAPDH was used as an endogenous control. The relative gene expression was evaluated using the ΔΔCt method.

### 2.11 Strep pull‐down assay

HeLa S3/Strep‐HA‐DESI1, HeLa S3/Strep‐HA‐FoxM1, and HeLa S3/Strep‐HA‐FoxM1/FLAG‐DESI1 cells were treated with 4 μg/mL Dox for 48 h and cultured in the presence of 5 μM STLC during the final 16 h. Cells were harvested using mitotic shake‐off and lysed with RIPA buffer containing 50 mM HEPES‐NaOH (pH 7.4), 150 mM NaCl, 0.1% SDS, 0.5% deoxycholate, 1% Triton X‐100, 4 mM EDTA‐NaOH, 2 μg/mL aprotinin, 0.8 μg/mL pepstatin A, 2 μg/mL leupeptin, 0.5 mM EGTA‐KOH (pH 8.0), and 1 mM PMSF for 10 min on ice. The lysates were then incubated with Strep‐Tactin 4Flow beads (2‐5010‐002, IBA Lifesciences, Göttingen, Germany) for 2 h at 4°C. The beads were washed four times with RIPA buffer and subsequently incubated with SDS‐sample buffer for 3 min at 100°C.

### 2.12 CCK‐8 assay

A thousand cells were seeded in a 96‐well plate and treated with siRNA. After 24 h, cells were cultured with dimethyl sulfoxide (DMSO, 0.05%), 1.5 nM vincristine (Nippon Kayaku, Tokyo, Japan), 5 μM proTAME (HY‐124955, MedChemExpress, NJ, USA), or a combination of these for 3 days. Subsequently, cells were incubated with the water‐soluble tetrazolium salt, 2‐(2‐methoxy‐4‐nitrophenyl)‐3‐(4‐nitrophenyl)‐5‐(2,4‐isulfophen‐yl)‐2H‐tetrazolium, monosodium salt (WST‐8, Cell Counting Kit‐8, 343‐07623, Dojindo, Kumamoto, Japan) for 2 h. The absorbance of the formazan dye was measured at 450 nm. The absorbance of each sample was normalized to the mean absorbance of control cells (siCtrl, DMSO).

### 2.13 Statistics

Statistical analysis was performed using Microsoft Excel (Microsoft, Redmond, WA, USA), EZR software (Saitama Medical Center, Jichi Medical University, Saitama, Japan),^36^ and R software (v. 3.6.2; R Foundation for Statistical Computing, Vienna, Austria). The difference between the two datasets was analyzed using Student's *t*‐test or Welch's *t‐*test after an *F*‐test was used to analyze their variances. The difference among more than two datasets was analyzed using Tukey's post hoc test or the Games‐Howell multiple comparison test after Bartlett's test was used to analyze their variances.

## 3 RESULTS

### 3.1 DESI1 knockdown accelerates M‐phase progression in the enzyme activity‐dependent manner

**FIGURE 1:**
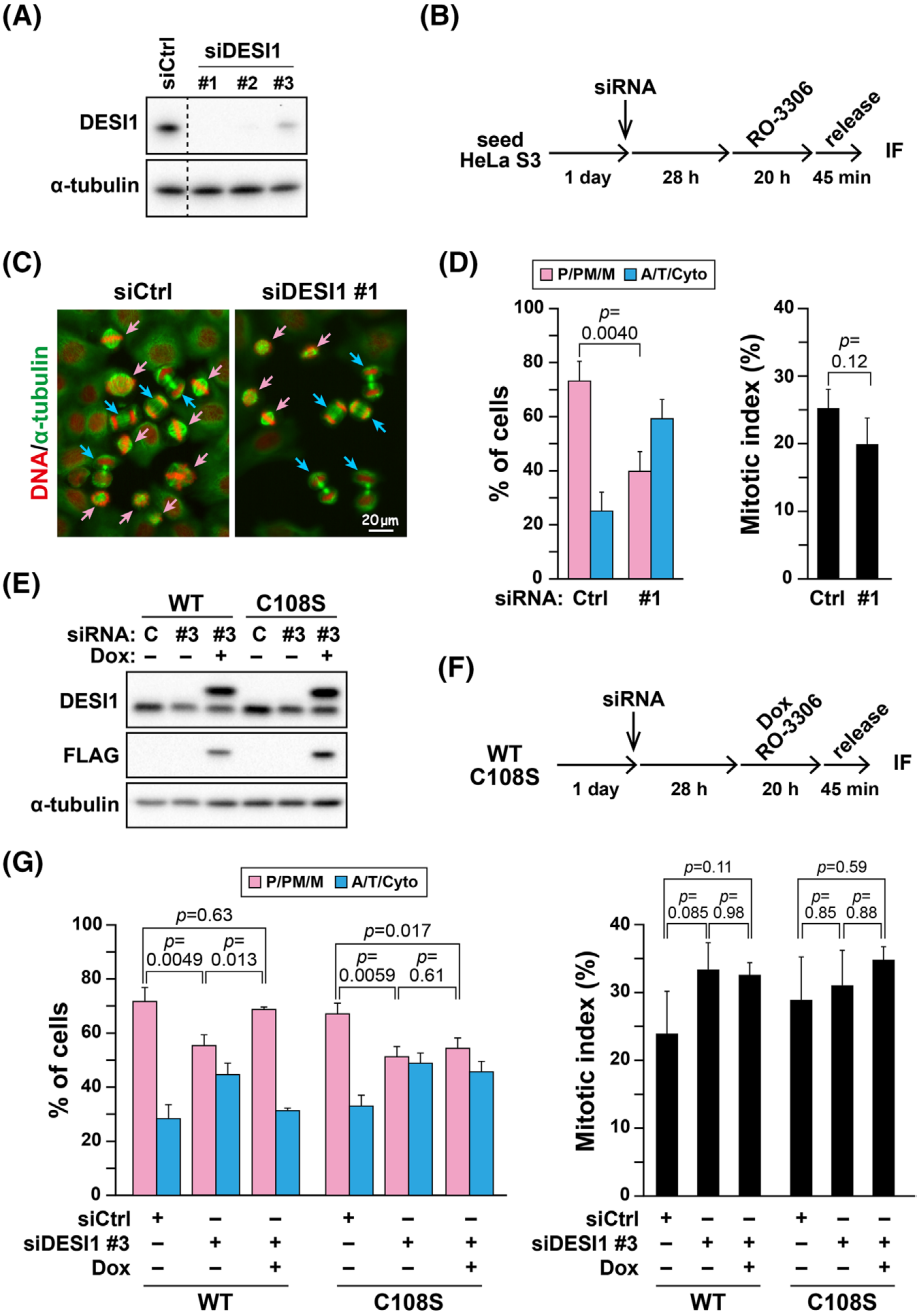
Acceleration of M‐phase progression due to DESI1 knockdown. (A) HeLa S3 cells underwent treatment with DESI1‐targeting (siDESI1) or non‐targeting (siCtrl) siRNAs for 48 h, followed by the analysis of whole‐cell lysates via Western blot analysis. (B–D) HeLa S3 cells were treated with siCtrl or siDESI1 #1 for 48 h and 6 μM RO‐3306 during the final 20 h. Subsequently, cells were cultured with fresh medium for 45 min without RO‐3306, and the fixed cells were immunostained using an anti‐α‐tubulin antibody and 1 μM Hoechst 33342. M‐phase progression was evaluated based on the morphologies of DNA and microtubules. In (B), a schematic depiction of the experimental method is shown. In (C), representative images are displayed. M‐phase cells were divided into two groups: before (P/PM/M, pink arrows) and after (A/T/Cyto, blue arrows) the anaphase onset. Scale bar, 20 μm. In (D), the ratios of each group (*n* > 188 per condition) and mitotic index (*n* > 1002 per condition), a ratio of the number of M‐phase cells to the total number of cells, are shown as the mean ± *SD* calculated from three independent experiments. *p*‐values were determined using the Student's *t*‐test. (E) HeLa S3/FLAG‐DESI1 WT and HeLa S3/FLAG‐DESI1 C108S cells were treated with siCtrl or siDESI1 #3 for 28 h. Then, expression of FLAG‐DESI1 WT or FLAG‐DESI1 C108S was induced by treatment with 0.4 μg/mL or 0.85 μg/mL doxycycline (Dox), respectively, for an additional 20 h. Whole‐cell lysates were analyzed by Western blot analysis. (F, G) HeLa S3/FLAG‐DESI1 WT and HeLa S3/FLAG‐DESI1 C108S cells were treated with siCtrl or siDESI1 #3 for 48 h and with 6 μM RO‐3306 during the final 20 h in the presence of Dox (0.4 μg/mL for WT; 0.85 μg/mL for C108S). Subsequently, cells were cultured in fresh medium for 45 min without RO‐3306. M‐phase cells were examined for mitotic subphases as described in (C). In (F), a schematic depiction of the experimental method is shown. M‐phase progression (*n* > 190 per condition) and mitotic index (*n* > 1002 per condition) were evaluated as described in (D). The results are shown in (G) as the mean ± *SD* calculated from three independent experiments. *p*‐values were determined using Tukey's multiple comparisons test.

Similar to SENP family proteins, deregulation of DESI1 is hypothesized to be associated with cancer, but the role of DESI1 in most cellular events remains largely unexplored. Given that proper chromosome segregation is crucial to preventing cancer development and malignancy, we examined the role of DESI1 in M‐phase progression. The reversible CDK1 inhibitor RO‐3306 halts cell cycle progression at the G2/M border, and upon its removal, approximately 30% of cells synchronously resume cell division. By classifying M‐phase cells into categories before (P/PM/M) or after (A/T/Cyto) the onset of anaphase, we can evaluate M‐phase progression. HeLa S3 cells were treated with siRNAs targeting three different regions of DESI1 (siDESI1 #1–3) and arrested at the G2/M border by treatment with RO‐3306 for 20 h. After removal of RO‐3306, cells were cultured with fresh medium for 45 min and analyzed for M‐phase progression (Figure 1A,B). Classification of M‐phase cells revealed that 74.2% of non‐targeting siRNA (siCtrl)–treated cells had not yet segregated their chromosomes, i.e., the cells were before the anaphase onset (Figure 1C, siCtrl, pink arrows, D, P/PM/M), while 25.8% of cells had done so (blue arrows). In stark contrast, more than half of the DESI1 knockdown cells had already segregated chromosomes (Figure 1C, siDESI1 #1, blue arrows, D, A/T/Cyto), suggesting that DESI1 knockdown accelerates M‐phase progression. The similar levels of the mitotic index indicated no difference in cell cycle synchronization between control and DESI1 knockdown cells. Other siRNAs targeting different regions (siDESI1 #2 and siDESI1 #3) yielded similar results (Figure S2).

To rule out the possibility of off‐target effects from siRNA treatment, we conducted knockdown‐rescue experiments using a cell line (HeLa S3/FLAG‐DESI1 WT) capable of doxycycline (Dox)‐inducible expression of FLAG‐tagged wild‐type DESI1. Following treatment with siRNA targeting the 3′‐untranslated region (3′‐UTR, #3) of the DESI1 mRNA, FLAG‐tagged wild‐type DESI1 expression was induced by Dox treatment. Synchronization experiment showed that DESI1 knockdown resulted in an increase in cells after the anaphase onset, and this effect was suppressed by the re‐expression of wild‐type DESI1 at levels similar to endogenous DESI1 (Figure 1E–G), thereby ruling out the off‐target effects of siRNA treatment. In sharp contrast, the catalytically inactive DESI1‐C108S mutant (HeLa S3/FLAG‐DESI1‐C108S) did not prevent the acceleration of M‐phase progression caused by DESI1 knockdown (Figure 1E–G). These results suggest that DESI1 plays a crucial role in M‐phase progression in a manner that is dependent on its catalytic activity.

### 3.2 Overexpression of DESI1 delays M‐phase progression

**FIGURE 2:**
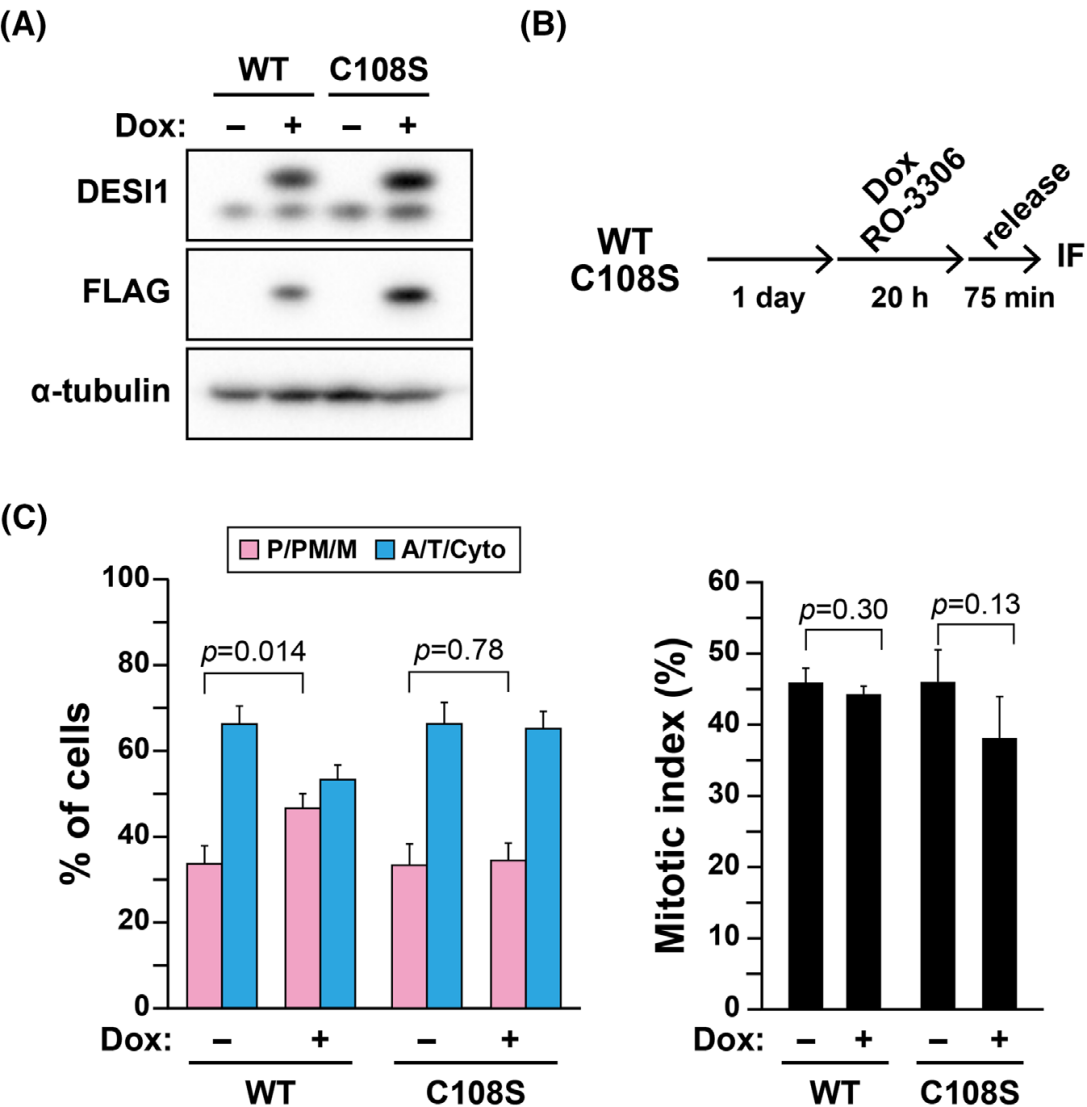
M‐phase progression is delayed by DESI1 overexpression. (A) HeLa S3/FLAG‐DESI1 WT and HeLa S3/FLAG‐DESI1 C108S cells were treated with 2 μg/mL and 4.3 μg/mL Dox for 20 h, respectively. Whole‐cell lysates were then analyzed using Western blot analysis. (B, C) HeLa S3/FLAG‐DESI1 WT and HeLa S3/FLAG‐DESI1 C108S cells were cultured with Dox at the concentrations described in (A) in the presence of 6 μM RO‐3306 for 20 h. After a 75‐min release from RO‐3306, cells were fixed and stained for α‐tubulin and DNA. A schematic depiction of the experimental method is shown in (B). M‐phase progression was evaluated as described in Figure 1C,D. The percentages of each group (*n* > 329 per condition) and the mitotic index (*n* > 1009 per condition) are shown as the mean ± *SD* calculated from three independent experiments. *p*‐values were determined using the Student's *t‐*test.

Overexpression of DESI1 is observed in certain cancer types (Figure S1). Consequently, we examined the impact of DESI1 overexpression on cell division. HeLaS3/FLAG‐DESI1 WT and HeLaS3/FLAG‐DESI1 C108S cells were treated with Dox for overexpression and synchronized with RO‐3306 (Figure 2A,B). While 66.3% of control cells segregated chromosomes at 75 min after release from RO‐3306 (Figure 2C, WT, Dox−), only 53.3% of wild‐type DESI1 overexpressing cells (WT, Dox+) did so. Conversely, overexpression of the catalytically inactive DESI1 C108S did not affect M‐phase progression, even when expressed at slightly higher levels than wild‐type DESI1. These results suggest that DESI1 overexpression may influence M‐phase progression.

### 3.3 Role of DESI1 is distinct from SENP1

**FIGURE 3:**
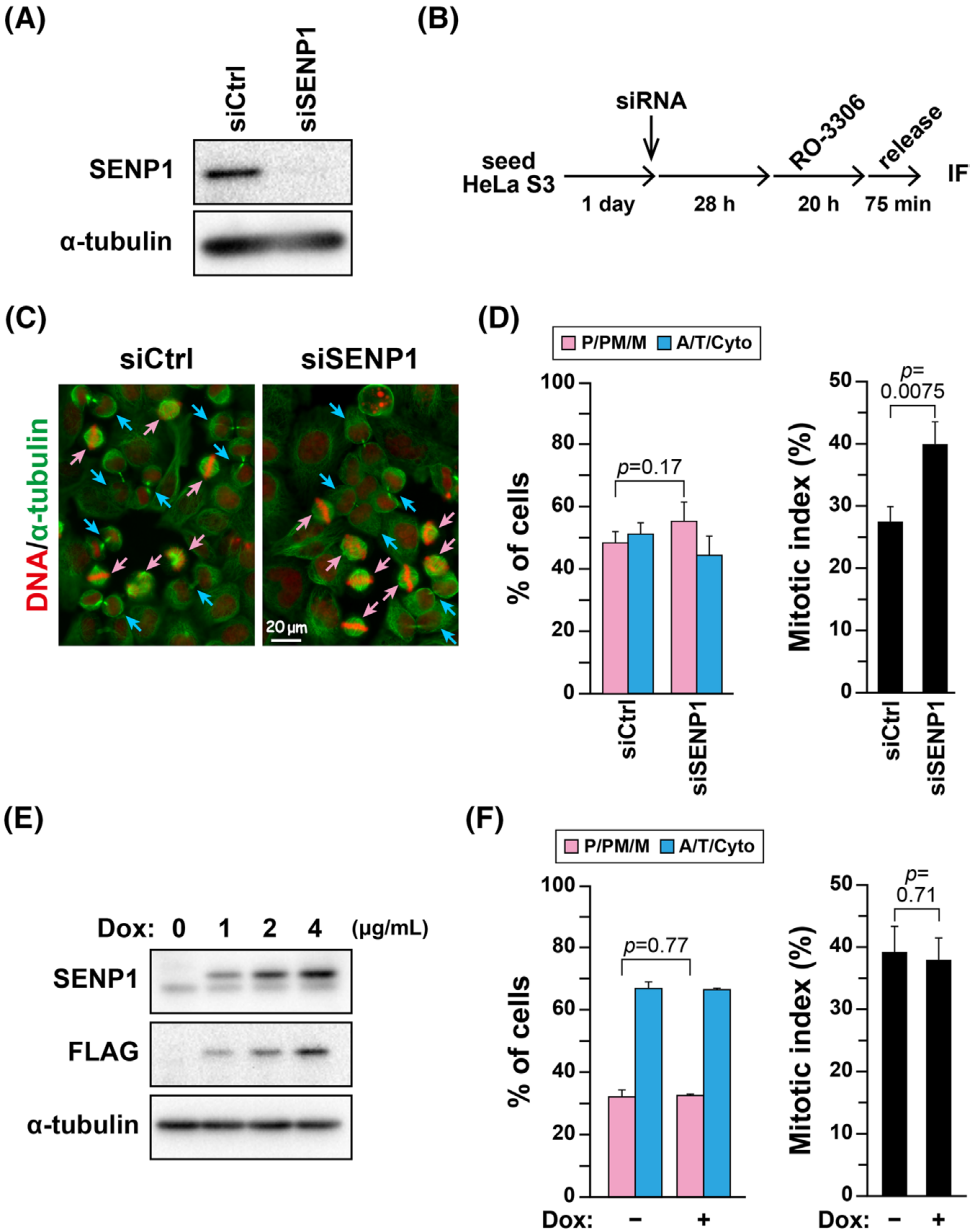
Delay in M‐phase progression due to SENP1 knockdown. (A) HeLa S3 cells were treated with either siCtrl or SENP1‐targeting siRNA for 48 h, and whole‐cell lysates were analyzed using Western blot analysis. (B–D) HeLa S3 cells were treated with siCtrl or siSENP1 for 48 h and cultured with 6 μM RO‐3306 during the final 20 h. The cells were then cultured without RO‐3306 for an additional 75 min. M‐phase progression was evaluated as described in Figure 1. A schematic depiction of the experimental method is shown in (B). Representative images are displayed in (C). DNA (red), microtubules (green). Scale bar, 20 μm. The results are shown as the mean ± *SD* calculated from three independent experiments (left graph, *n* > 269; right graph, *n* > 1044 per condition). *p*‐values were determined using the Student's *t‐*test. (E) HeLa S3/FLAG‐SENP1 cells were treated with 0–4 μg/mL Dox for 24 h, and whole‐cell lysates were analyzed using Western blot analysis. (F) HeLa S3/FLAG‐SENP1 cells were simultaneously incubated with 2 μg/mL Dox and 6 μM RO‐3306 for 20 h. The cells were then cultured without RO‐3306 for 60 min. M‐phase progression was evaluated as described in Figure 1. The results are shown as the mean ± *SD* calculated from three independent experiments (left graph, *n* > 362; right graph, *n* > 1005 per condition). *p*‐values were determined using the Student's *t‐*test.

SUMOylation with SUMO1 and SUMO2/3 of key cell division regulators participates in regulating their functions.^18^, ^19^, ^20^, ^21^, ^22^, ^23^ SENP family proteins have been extensively studied due to their crucial role in the deSUMOylation of proteins.^24^ SENP1 and SENP2 catalyze the deSUMOylation of SUMO1 and SUMO2/3. However, the other SENPs do not catalyze SUMO1 deSUMOylation.^37^ SENP2 knockdown does not cause any defects in cell division, whereas SENP1 knockdown prolongs the metaphase duration.^38^ Therefore, we compared the role of SENP1 with that of DESI1 in cell division. SENP1 was knocked down, and the impact on M‐phase progression was examined using RO‐3306. Contrary to DESI1, the knockdown of SENP1 did not accelerate M‐phase progression; instead, it appeared to delay it, albeit not significantly (Figure 3A–D). The mitotic index notably increased in SENP1 knockdown, suggesting a potential prolongation of the entire M‐phase duration. Additionally, SENP1 overexpression demonstrated no effect on M‐phase progression (Figure 3E,F), as previously reported.^38^ These results imply that the role of DESI1 in M‐phase progression is distinct from that of SENP1. Moreover, SENP1 overexpression, unlike its knockdown, did not affect the mitotic index. One possibility is that SENP1 at the endogenous level may sufficiently and properly deSUMOylate cell division–related SENP1 substrates with high substrate specificity, which could explain why SENP1 knockdown but not overexpression may affect cell division via regulating SUMOylation levels of these substrates.

### 3.4 DESI1 contributes to faithful chromosome segregation

**FIGURE 4:**
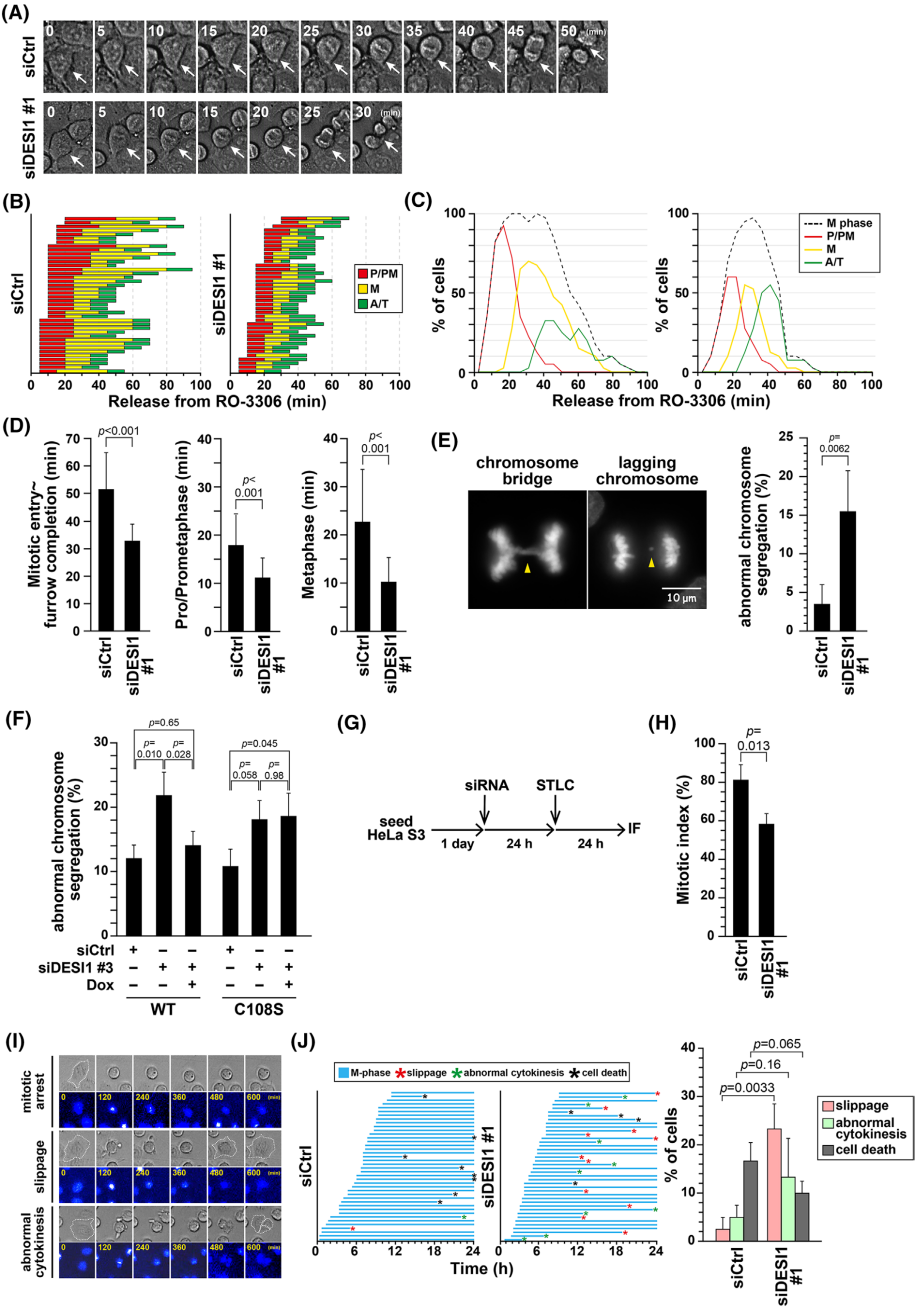
DESI1 is essential for accurate chromosome segregation. (A–D) HeLa S3 cells underwent treatment with siCtrl or siDESI1 #1 for 48 h and 6 μM RO‐3306 for the final 20 h. Following release from RO‐3306, cells were observed under time‐lapse analysis in the presence of 0.1 μM Hoechst 33342 for 180 min at 5‐min intervals. In (A), a series of representative images are displayed. Arrows point to individual cells. In (B), the length of mitotic subphases (red, P/PM; yellow, M; green, A/T) for each cell is depicted (*n* = 40). In (C), the proportions of each subphase at the specified time points are graphed. The dashed black lines represent the percentages of mitotic cells. In (D), the time spans from M‐phase entry to furrow compression, prophase/prometaphase, and metaphase are presented as the mean ± *SD* (*n* = 40). *p*‐values were calculated using Welch's *t*‐test. (E) Cells with DESI1 knockdown were synchronized as outlined in Figure 1B. Images of representative cells exhibiting chromosome bridge and lagging chromosome are displayed, and their percentages are graphed as the mean ± *SD* calculated from three independent experiments (*n* = 50 per condition). *p*‐values were calculated using the Student's *t*‐test. Scale bar, 10 μm. (F) DESI1 was depleted from HeLa S3/FLAG‐DESI1 WT and HeLa S3/FLAG‐DESI1 C108S cells and re‐expressed via Dox treatment (WT, 0.4 μg/mL; C108S, 0.85 μg/mL). The cells were synchronized as outlined in Figure 1F. The mean values ±*SD* were calculated from three independent experiments (*n* ≥ 50 per condition). *p*‐values were calculated using Tukey's multiple comparisons test. (G, H) HeLa S3 cells underwent treatment with siCtrl or siDESI1 #1 for 48 h, and during the final 24 h cells were synchronized by treatment with 7.5 μM STLC. Subsequently, cells were fixed with 100% methanol. A schematic representation of the experimental method is displayed in (G). In (H), the mitotic index is graphed as the mean ± *SD* calculated from three independent experiments (*n* > 500 per condition). *p*‐values were calculated using Student's *t‐*test. (I, J) HeLa S3 cells were treated with siCtrl or siDESI1 #1 for 24 h and then monitored by time‐lapse analysis at 20‐min intervals for 24 h in the presence of 50 ng/mL nocodazole and 0.1 μM Hoechst 33342. In (I), representative images of mitotic arrest, slippage, and abnormal cytokinesis are displayed. In (J), the duration of M phase of individual cell is shown on the left (*n* = 40). Red, green, and black asterisks indicate mitotic slippage, abnormal cytokinesis, and cell death, respectively. On the right, the percentage of cells exhibiting slippage, abnormal cytokinesis, and cell death are shown as the mean ± *SD* calculated from three independent experiments (*n* = 40). *p*‐values were calculated using Student's *t‐*test.

Given that DESI1 knockdown accelerated M‐phase progression, we investigated its effect on chromosome segregation using time‐lapse analysis. Consistent with the results obtained by immunofluorescence staining of fixed cells (Figure 1), the M‐phase duration of DESI1 knockdown cells was shortened (Figure 4A). For quantitative analysis, M phase was divided into three subphases: from the start of mitotic cell rounding to just before chromosome alignment (P/PM), during chromosome alignment (M), and from the onset of chromosome segregation to the completion of cleavage furrow ingression (A/T). The progression of M phase for each cell was plotted in Figure 4B, and the ratio of each mitotic subphase at each time point was plotted in Figure 4C. The M‐phase duration was 51.4 min in control cells, while DESI1 knockdown reduced it to 32.8 min (Figure 4B,D). Moreover, the P/PM duration decreased from 17.9 to 11.1 min, and the metaphase duration decreased from 22.8 to 10.3 min (Figure 4D). The A/T peak was elevated in DESI1 knockdown cells, indicating synchronization in the anaphase due to the shortening of each mitotic subphase (Figure 4C). Additionally, the ratio of abnormal chromosome segregation, such as chromosome bridge and lagging chromosome, increased in DESI1 knockdown cells from 3.5% to 15.5% compared to control cells (Figure 4E), indicating that DESI1 is essential for faithful chromosome segregation. The abnormal chromosome segregation caused by DESI1 knockdown (siDESI1 #3) was rescued by the re‐expression of wild‐type but not catalytically inactive DESI1 (Figure 4F). These results suggest that the catalytic activity of DESI1 is necessary for faithful chromosomal segregation.

S‐trityl‐L‐cysteine (STLC), an Eg5 inhibitor, induces the formation of a monopolar spindle by inhibiting centrosome separation. STLC treatment impedes M‐phase progression by activating the SAC, leading to the accumulation of cells with a monopolar spindle over time. To investigate whether DESI1 influences STLC‐induced cell cycle arrest, DESI1 knockdown cells were treated with STLC (Figure 4G). Although the mitotic index was 81.7% in control cells, it decreased to 58.7% in DESI1 knockdown cells (Figure 4H), indicating mitotic slippage, i.e., M‐phase exit without cytokinesis, despite the presence of STLC. Furthermore, time‐lapse imaging directly revealed that DESI1 knockdown cells exit mitosis even in the presence of nocodazole (Figure 4I,J), suggesting that DESI1 knockdown cells undergo mitotic slippage in the presence of STLC or nocodazole.

### 3.5 DESI1 contributes to chromosomal localization of Aurora B

Mitotic slippage in DESI1 knockdown cells (Figure 4) suggests a potential involvement of DESI1 in the regulation of the SAC. Aurora B, in addition to its role in correcting improper kinetochore‐microtubule attachments,^39^, ^40^ also plays a part in SAC by facilitating MPS1 recruitment to unattached kinetochores and by guiding the assembly of the mitotic checkpoint complex (MCC).^6^, ^7^ Aurora B forms the chromosome passenger complex with INCENP, Survivin, and Borealin. It localizes on the chromosomes during prometaphase and metaphase and subsequently relocates to the spindle midzone.^41^

**FIGURE 5:**
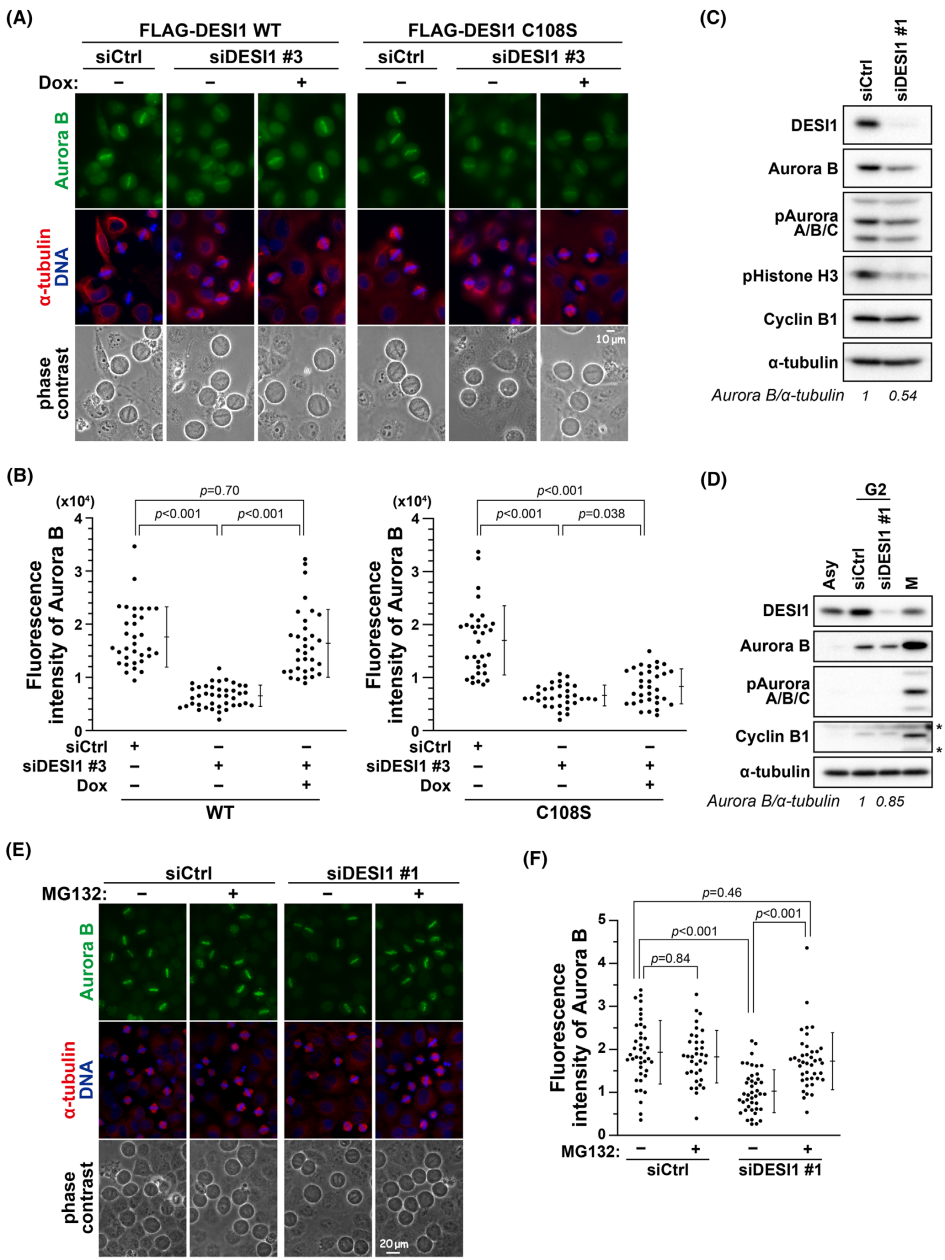
Reduction of Aurora B localization on chromosomes by DESI1 knockdown. (A, B) HeLa S3/FLAG‐DESI1 WT and HeLa S3/FLAG‐DESI1 C108S cells were treated with siCtrl or siDESI1 #3 for 48 h in the presence of Dox (WT, 0.4 μg/mL; C108S, 0.85 μg/mL). In the final 20 h, cells were treated with 6 μM RO‐3306. Following a 30‐min release from RO‐3306, cells were fixed with 4% formaldehyde in PBS (−) and stained for α‐tubulin (red), Aurora B (green), and DNA (blue). Representative images are displayed in (A). Scale bar, 10 μm. The fluorescence intensity of Aurora B on chromosomes was quantified using ImageJ, and the results are shown as the mean ± *SD* in (B) (*n* ≥ 30 per condition). *p*‐values were calculated using the Games‐Howell multiple comparison test. (C) HeLa S3 cells were treated with siCtrl or siDESI1 #1 for 48 h and cultured in the presence of 4 mM thymidine for the final 24 h. After thymidine removal, cells were cultured for 9 h and then treated with 6 μM RO‐3306 for an additional 10 h. Following the removal of RO‐3306, cells were treated with 5 μM STLC for 1 h. Subsequently, mitotic cells were collected and analyzed by Western blot analysis. (D) HeLa S3 cells were treated with siRNA and thymidine as outlined in (C). After thymidine removal, G2 and M‐phase cells were obtained after 9‐h culture without any drug (G2) and 16‐h culture with 5 μM STLC (M), respectively, and subjected to Western blot analysis. (E, F) HeLa S3 cells were treated with siCtrl or siDESI1 #1 for 48 h and 6 μM RO‐3306 during the final 20 h. After a 5‐min culture without RO‐3306, cells were treated with 40 μM MG‐132 for an additional 25 min, fixed using 4% formaldehyde in PBS (−), and stained for α‐tubulin (red), Aurora B (green), and DNA (blue). Representative images are displayed in (E). Scale bar, 20 μm. The fluorescence intensity of Aurora B on chromosomes was quantified, and the results are shown as the mean ± *SD* (*n* ≥ 35 per condition). *p*‐values were calculated using Tukey's multiple comparisons test.

To determine whether Aurora B was implicated in the phenotype caused by DESI1 knockdown, we examined the chromosomal localization of Aurora B in DESI1 knockdown cells. HeLa S3/FLAG‐DESI1 WT cells were treated with RO‐3306, and 30 min post‐release of RO‐3306, cells were fixed and stained for Aurora B. At this time, metaphase cells were frequently observed. Control cells exhibited Aurora B localization on the chromosomes in metaphase (Figure 5A, siCtrl, Dox–, B). In contrast, DESI1 knockdown (siDESI1 #3) reduced the localization of Aurora B on chromosomes, which was rescued by re‐expression of wild‐type DESI1 but not the catalytically inactive DESI1 C108S mutant (Figure 5A,B). Consistently, Aurora B localization on the midzone and the midbody appears to be reduced after a 60‐min release from RO‐3306 (Figure S3). Furthermore, Western blot analysis revealed that the level of Aurora B was decreased in M phase upon DESI1 knockdown (Figure 5C), suggesting that the delocalization of Aurora B is attributed to the decrease in Aurora B protein levels. Since Aurora B is expressed in the G2 phase, G2‐synchronized cells treated with thymidine were examined for Aurora B levels. Asynchronized cells showed only a faint band, and Aurora B level was increased in G2‐synchronized control cells. Upon DESI1 knockdown, Aurora B level was slightly decreased compared to control cells in G2 (Figure 5D). To investigate whether DESI1 was involved in Aurora B degradation, DESI1 knockdown cells were treated with the proteasome inhibitor MG132. MG132 treatment restored Aurora B localization on chromosomes at metaphase (Figure 5E,F). Notably, MG132 treatment did not increase Aurora B localization in control cells (Figure 5E,F, siCtrl). These results suggest that DESI1 knockdown reduces Aurora B stability. Collectively, the acceleration of M‐phase progression caused by DESI1 knockdown may be due to SAC attenuation via a reduction of Aurora B levels.

### 3.6 DESI1 regulates FoxM1 transcriptional activity

**FIGURE 6:**
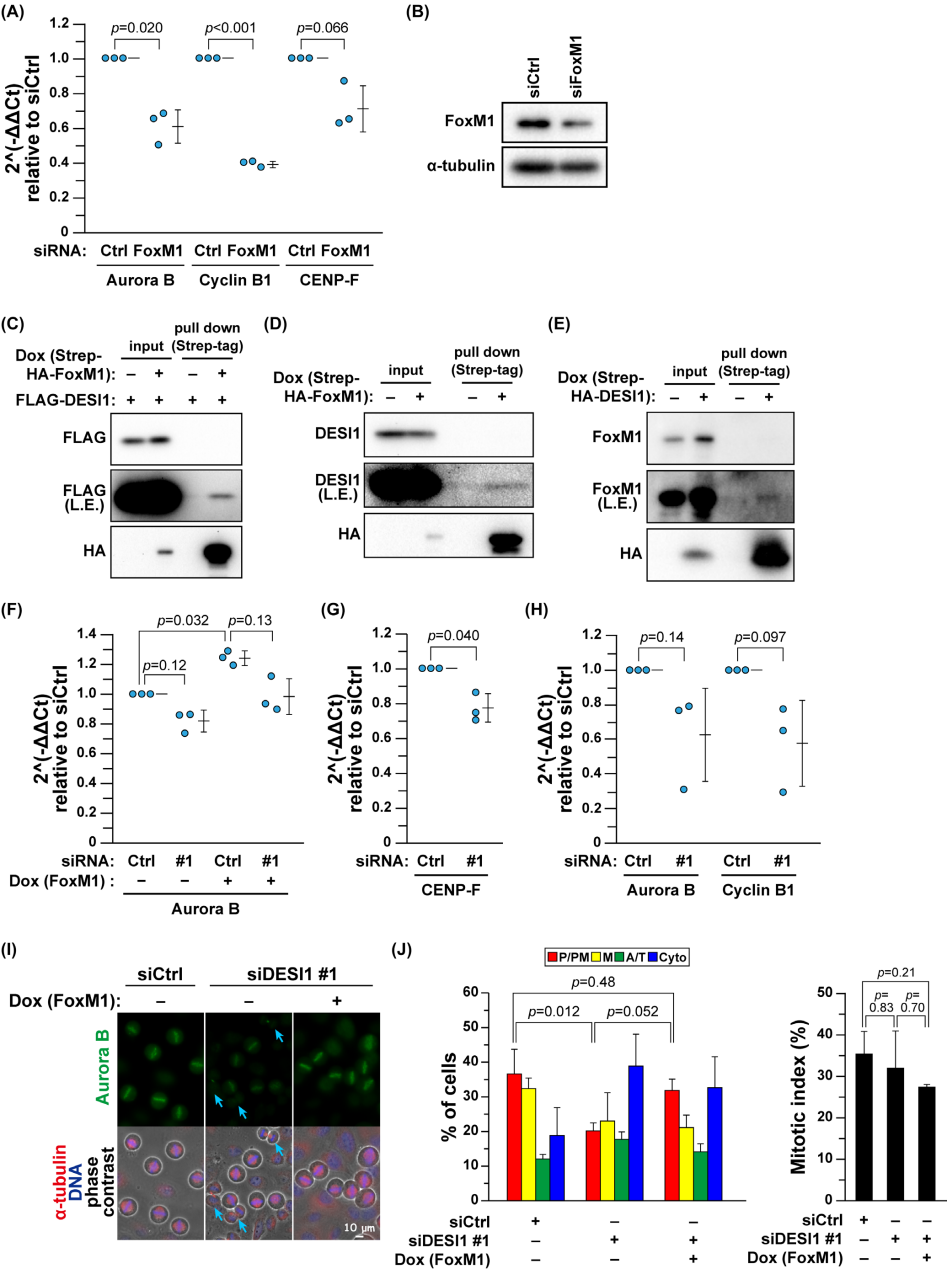
The reduction in mRNA levels of FoxM1 target genes upon DESI1 knockdown. (A, B) HeLa S3 cells were transfected with siCtrl or siFoxM1. After a 6‐h incubation, cells were treated with 5 μM STLC for 16 h. The mitotic cells were harvested via mitotic shake‐off, and the mRNA levels of Aurora B, cyclin B1, and CENP‐F were analyzed by real‐time PCR. In (A), the fold changes in mRNA levels are expressed relative to siCtrl, and the results are displayed as the mean ± *SD* calculated from three independent experiments. *p*‐values were calculated using Welch's *t‐*test. Knockdown was confirmed by Western blot analysis in (B). (C–E) HeLa S3/Strep‐HA‐FoxM1/FLAG‐DESI1 cells (C), HeLa S3/Strep‐HA‐FoxM1 cells (D), and HeLa S3/Strep‐HA‐DESI1 cells (E) were treated with 4 μg/mL Dox for 48 h and 5 μM STLC during the last 16 h. The mitotic cells were harvested via mitotic shake‐off and solubilized with RIPA buffer. The lysates were subjected to the pull‐down assay with Strep‐Tactin resin, and co‐precipitated protein was detected by Western blot analysis. (F) HeLa S3/Strep‐HA‐FoxM1 cells were transfected with siCtrl or siDESI1 (#1) and cultured in medium containing 4 μg/mL Dox for 22 h. During the last 16 h, 5 μM STLC was added. The mitotic cells were harvested via mitotic shake‐off, and the mRNA levels of Aurora B were analyzed by real‐time PCR. The fold changes in mRNA levels are expressed relative to siCtrl (Dox−), and the results are displayed as the mean ± *SD* calculated from three independent experiments. *p*‐values were calculated using Games‐Howell multiple comparison test. (G) The cells were treated with siDESI1 (#1), and mRNA was obtained as outlined in (A). The mRNA levels of CENP‐F were analyzed by real‐time PCR. (H) HeLa S3 cells were transfected with siCtrl or siDESI1 #1. After a 24‐h incubation, cells were cultured in a medium containing 4 mM thymidine for 24 h, followed by an additional 9‐h culture without thymidine. The mRNA levels of Aurora B and cyclin B1 were analyzed by real‐time PCR. The fold changes in mRNA levels are expressed relative to siCtrl, and the results are displayed as the mean ± *SD* calculated from three independent experiments. *p*‐values were calculated using Welch's *t‐*test. (I, J) HeLa S3/Strep‐HA‐FoxM1 cells were transfected with siCtrl or siDESI1 #1, and after a 6‐h culture, the medium was replaced with a fresh one, followed by a 22‐h culture. The cells were then incubated with 4 μg/mL Dox and 6 μM RO‐3306 for an additional 20 h. After a 45‐min release from RO‐3306, the cells were fixed and immunofluorescence staining was performed as described in Figure 5, In (I), representative images are displayed. Scale bar, 10 μm. The blue arrows point to cells that have progressed beyond the anaphase. In (J), M‐phase progression was evaluated as described in Figure 1. M‐phase cells were divided into four groups: prophase/prometaphase (P/PM), metaphase (M), anaphase/telophase (A/T), and cytokinesis (Cyto). The results are shown as the mean ± *SD* calculated from three independent experiments (left graph, *n* > 272; right graph, *n* > 1002 per condition). *p*‐values were determined using the Tukey's multiple comparisons test.

Transcription in addition to degradation regulates the Aurora B protein level. The Forkhead transcription factor FoxM1 is crucial for cell division as it transcriptionally regulates numerous genes that are crucial for accurate chromosome segregation, including Aurora B kinase,^42^, ^43^, ^44^ cyclin B1,^43^ and CENP‐F.^43^ Indeed, an mRNA level reduction of these genes in M phase was confirmed upon FoxM1 knockdown (Figure 6A,B). A pull‐down assay demonstrated an interaction between the overexpressed DESI1 and FoxM1 (Figure 6C). Endogenous DESI1 and FoxM1 were coimmunoprecipitated with Strep‐HA‐tagged FoxM1 (Figure 6D) and DESI1 (Figure 6E), respectively. Expectedly, DESI1 knockdown downregulated the mRNA levels of Aurora B (Figure 6F). Notably, DESI1 knockdown suppressed the increase in the Aurora B mRNA level induced by FoxM1 overexpression [Figure 6F, Dox(+) vs. Dox(+) & #1]. DESI1 knockdown reduced the mRNA levels of CENP‐F in M phase (Figure 6G) and both Aurora B and cyclin B1 in G2 phase (Figure 6H). These results suggest that DESI1 knockdown may downregulate FoxM1 transcriptional activity. Moreover, FoxM1 overexpression in DESI1‐knockdown cells mitigated the DESI1 knockdown–induced decrease in chromosomal Aurora B localization (Figure 6I) and the accelerated M‐phase progression (Figure 6J), suggesting that DESI1 maintains chromosomal Aurora B localization by regulating FoxM1 transcription activity. Altogether, DESI1 ensures mitotic fidelity by controlling the SAC activity by maintaining Aurora B levels on chromosomes.

### 3.7 DESI1 expression levels are associated with cancer

**FIGURE 7:**
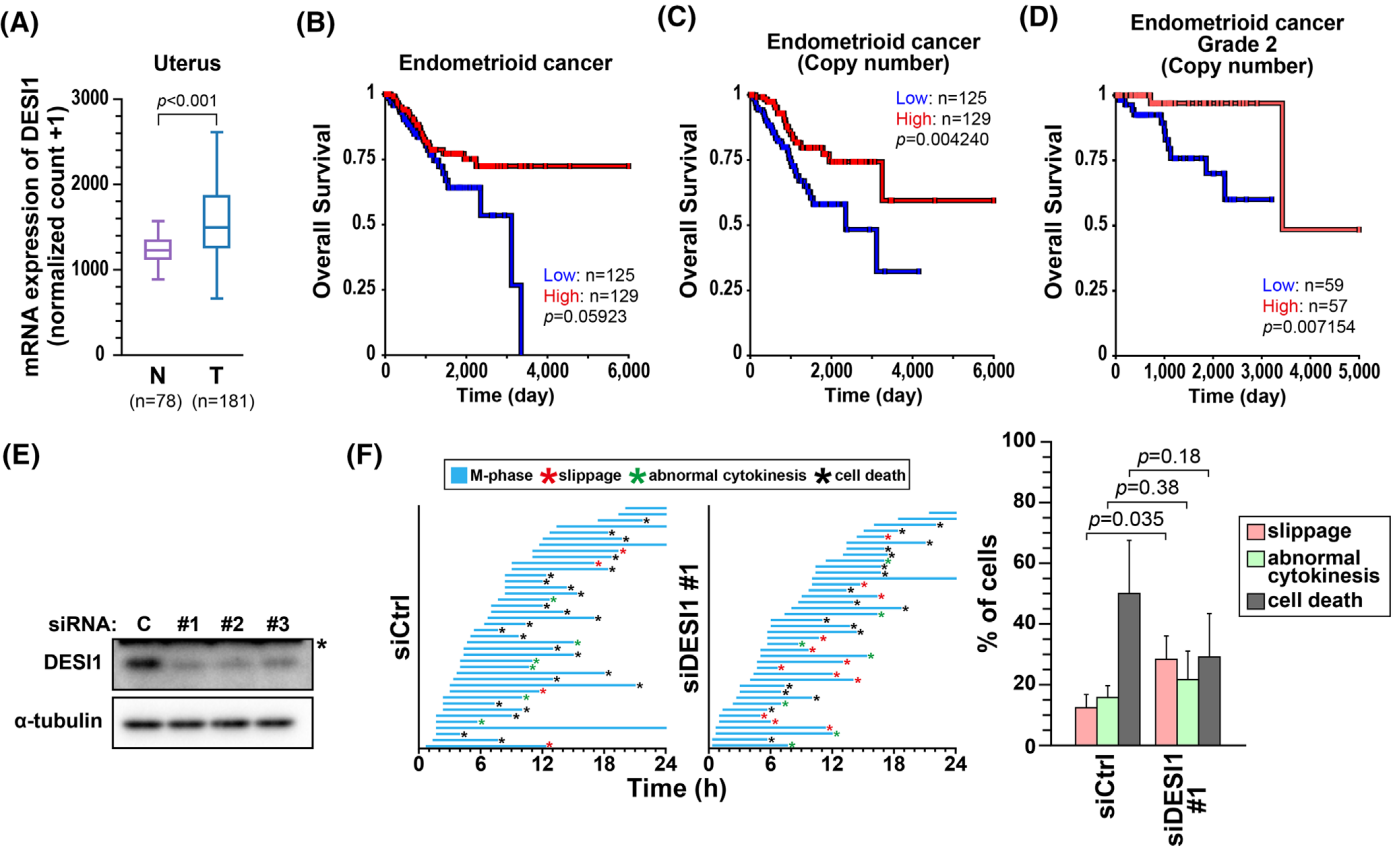
Deregulation of DESI1 expression is associated with overall survival of patients with cancer. (A) The relative mRNA expression of DESI1 in normal uterine tissue (N) and tumor [uterine corpus endometrioid carcinoma, (T)] in TCGA datasets are shown. *p*‐values were calculated by Welch's *t‐*test. (B–D) Endometrioid cancer patients in TCGA datasets were divided into two groups based on DESI1 expression level in (B) or its copy number in (C) and (D) (D; endometrioid cancer grade 2), and Kaplan–Meier curves for overall survival are shown. The patients in top quartile and bottom quartile, in terms of protein expression in (B) and copy number in (C), were compared with respect to overall survival. In (D), the patients were divided according to median copy numbers. (E) HEC‐1‐A cells were treated with siCtrl or siDESI1 #1–3 for 48 h, and whole‐cell lysates were analyzed by Western blot analysis. (F) HEC‐1‐A cells were treated with siCtrl or siDESI1 #1. After the 24‐h treatment, time‐lapse analysis was performed in the presence of 50 ng/mL nocodazole and 0.1 μM Hoechst 33342 for 24 h at 20‐min intervals. In (F), the duration of M phase of individual cell is shown in Figure 4J (*n* = 40). On the right, the percentage of cells exhibiting slippage, abnormal cytokinesis, and cell death are shown as the mean ± *SD* calculated from three independent experiments (*n* = 40). *p*‐values were calculated using Student's *t‐*test.

Both DESI1 knockdown (Figures 1 and 4) and overexpression (Figure 2) caused cell division defects. We compared the expression levels of DESI1 between normal (GTEx data) and cancer tissues and investigated the association of its levels with the prognosis of patients with cancer using the Cancer Genome Atlas datasets via UCSC Xena (https://xena.ucsc.edu) because defects in cell division cause cancer development and malignant progression (Figures 7 and S4). Pan‐cancer and some cancer types, including pancreatic cancer, melanoma, and thyroid cancer, showed DESI1 upregulation in cancer cells compared with normal cells (Figure S4A–D). Kaplan–Meier survival analysis revealed that an increased expression of DESI1 is associated with a poorer prognosis in patients with these cancer types (Figure S4E–H). Interestingly, although DESI1 expression levels are higher in endometrioid cancer cells than in a normal uterus, a low copy number of the DESI1 gene, especially in grade 2 patients, is significantly associated with poor prognosis (Figure 7A–D). Therefore, we investigated the requirement of DESI1 for cell division fidelity in human endometrioid cancer HEC‐1‐A cells by assessing SAC activity. Time‐lapse analysis of HEC‐1‐A cells in the presence of nocodazole revealed that DESI1 knockdown increased miotic slippage and decreased cell death instead, compared with control knockdown (Figure 7E,F). These results suggest that SAC activity attenuation, and thus the resulting chromosomal instability, may be one of the contributing factors to endometrioid cancer development.

### 3.8 Vincristine resistance in cells with low DESI1 expression

**FIGURE 8:**
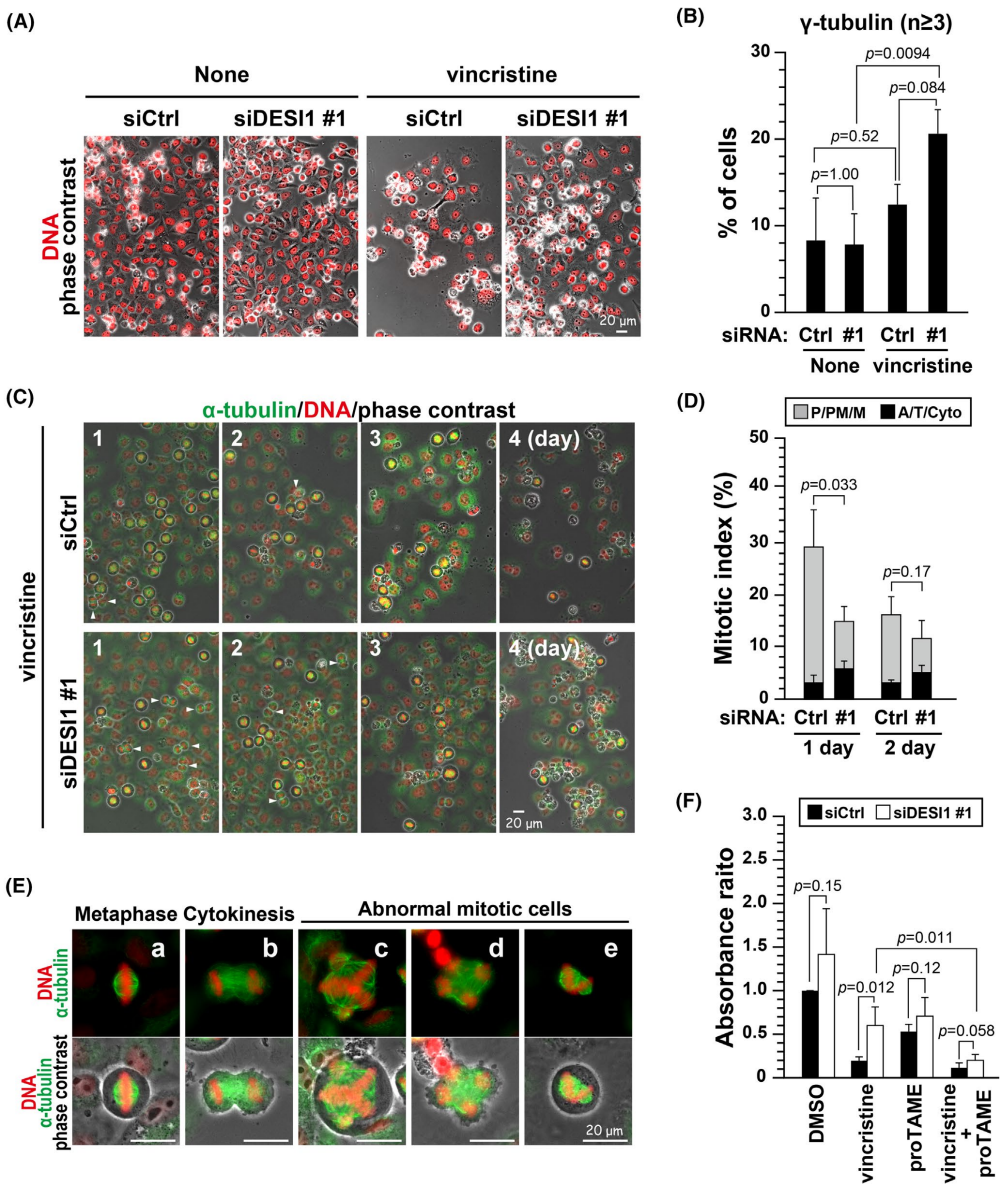
DESI1 knockdown results in decreased sensitivity to vincristine. (A–E) HeLa S3 cells were transfected with siCtrl or siDESI1 #1. Following a 24‐h culture, cells were exposed to 1.5 nM vincristine (VCR) for a period of 4 days. In (A), representative images are displayed. Scale bar, 20 μm. In (B), cells were immunostained for γ‐tubulin, and the number of centrosomes was counted. The graph depicts the percentage of cells with more than three centrosomes as the mean ± *SD* calculated from three independent experiments (*n* > 204). *p*‐values were calculated using Tukey's multiple comparisons test. In (C), cells were fixed and stained for α‐tubulin (green) and DNA (red) over a period of 1–4 days. Representative images are displayed. Scale bar, 20 μm. White arrowheads point to cells initiating chromosome segregation. In (D), the mitotic index is displayed, and mitotic cells were categorized into two groups: before (P/PM/M) and after (A/T/Cyto) the onset of anaphase. The ratios of the mitotic index (*n* > 1004 per condition) and each group (*n* > 208 per condition) are displayed as the mean ± *SD* calculated from three independent experiments. *p*‐values were calculated using the Student's *t‐*test. The statistical significance between siCtrl‐treated cells and siDESI1 (#1)‐treated cells for the ratio of cells in A/T/Cyto are as follows: *p* = .012 after a 1‐day culture; *p* = .025 after a 2‐day culture. In (E), the representative images of cells with normal metaphase (a), cytokinesis (b), and abnormal cells with multipolar spindle (c), multipolar cytokinesis (d), and two spindles (e) are shown. Scale bar, 20 μm. (F) HeLa S3 cells were treated with siDESI1 #1. After 24 h, cells were incubated with 1.5 nM VCR, 5 μM proTAME, or their combination for a duration of 3 days, and a CCK‐8 assay was performed. The absorbance ratio is expressed relative to control cells (siCtrl, DMSO), and the results are displayed as the mean ± *SD* calculated from five independent experiments. *p*‐values were calculated using the Student's *t‐*test or Welch's *t‐*test.

Microtubule‐targeting agents, including taxanes and vinca alkaloids, have been widely used in cancer chemotherapy. These agents arrest cells before the onset of anaphase via SAC activation. When SAC is inactivated by reducing Mad2 or BubR1, sensitivity to paclitaxel decreases,^45^, ^46^ suggesting that M‐phase arrest caused by SAC is required for its cytotoxicity.^47^ DESI1 knockdown attenuated SAC activity (Figures 4J and 7F); therefore, we examined whether DESI1 knockdown affected sensitivity to the vinca alkaloid vincristine (VCR). HeLa S3 cells were treated with control or DESI1‐targeting siRNA and then treated with VCR for 4 days. Microscopic analysis showed that more DESI1‐knockdown cells survived compared to control knockdown cells (Figure 8A). An increase in the number of centrosomes (γ‐tubulin) suggests that mitotic slippage occurs in DESI1‐knockdown cells (Figure 8B). However, the ratio of cells with an excess number of centrosomes was less than expected. VCR‐treated cells were fixed at consecutive 4‐day intervals and examined for progression to anaphase or later subphases (Figure 8C,D, A/T/Cyto). At 1 day of VCR treatment, the mitotic index was drastically reduced in DESI1‐knockdown cells, but the ratio of cells after the onset of anaphase (A/T/Cyto) was increased (*p* = .012), suggesting that the reduced mitotic index is due to mitotic slippage. Notably, severe defects in chromosome alignment were observed in DESI1 knockdown cells at 1 day of VCR treatment (Figure 8E; a and b, normal; c–e, abnormal). They include a multipolar spindle in metaphase (Figure 8E‐c), a multipolar cytokinesis (Figure 8E‐d), and two spindles in a cell (Figure 8E‐e), suggesting that survived cells upon DESI1 knockdown could give rise to induction of chromosomal instability. The CCK‐8 assay showed that DESI1 knockdown increased the number of cells compared to control cells (Figure 8F). VCR treatment exhibited strong cytotoxicity to control cells; however, about half of DESI1 knockdown cells survived after a 72‐h treatment with VCR. Interestingly, the combination of the APC/C inhibitor proTAME with vincristine significantly reduced the number of surviving cells. These results suggest that decreased DESI1 expression levels confer resistance to VCR in cancer cells, which may be overcome by combination with the APC/C inhibitor.

## 4 DISCUSSION

SUMOylation status, which plays significant roles in numerous cellular events including cell division, has been the subject of extensive research, particularly in the context of SENP family deSUMOylase proteins. However, other deSUMOylase proteins have been less well characterized. In this study, we explored the role of another deSUMOylase, DESI1, and discovered that it plays a crucial role in ensuring accurate chromosome segregation, distinct from the role of SENP1. DESI1 knockdown did not alter the overall cellular pattern of SUMO‐modified proteins,^26^ indicating that DESI1 fine‐tunes SUMOylation levels in the regulation of cell division. Data from the TCGA database revealed that deregulation of DESI1 is associated with a poorer cancer patient prognosis. These findings lay the groundwork for the development of novel therapeutic strategies.

In this study, we found that DESI1 knockdown expedited the progression of cell division (Figures 1 and 4). This could be attributed to more efficient formation of the mitotic spindle and chromosome alignment. However, this acceleration of cell division was accompanied by a defect in chromosome segregation (Figure 4E,F). This suggests that DESI1 knockdown leads to premature progression of cell division and underscores the role of DESI1 in ensuring faithful chromosome segregation. Time‐lapse imaging revealed a significant shortening of the metaphase duration (Figure 4A–D). The duration of metaphase primarily depends on the activity of the SAC, which ensures the proper attachment of kinetochores to microtubules.^5^ Mitotic slippage observed under time‐lapse imaging (Figure 4I,J) directly proved a reduction in SAC function by DESI1 knockdown. Therefore, a reduction in SAC function results in a premature onset of anaphase with errors in chromosome segregation.

Our findings revealed that DESI1 maintains Aurora B protein levels at the transcriptional level in addition to stabilizing Aurora B proteins. FoxM1 SUMOylation, which regulates its transcriptional activity, has been reported.^20^, ^21^, ^48^ Therefore, it is plausible that DESI1 knockdown reduces Aurora B protein levels by affecting FoxM1 SUMOylation levels. FoxM1 is SUMOylated at multiple sites, but the effects of SUMOylation on its transcriptional activity are controversial.^20^, ^21^, ^48^ A study using a FoxM1‐UBC9 fusion protein to facilitate SUMOylation and a SUMOylation‐deficient mutant showed that SUMO1 modification of FoxM1 is inhibitory, and the expression of the SUMOylation‐deficient mutant causes persistent cyclin B1 expression and delays M‐phase progression.^21^ Furthermore, FoxM1 SUMOylation promotes its degradation in a Cdh1‐dependent manner. If DESI1 knockdown increases SUMOylation levels of FoxM1 by SUMO1, a reduction in the transcription of FoxM1 target genes is expected. In fact, the transcription of Aurora B, cyclin B1, and CENP‐F was reduced in DESI1 knockdown cells (Figure 6). Additionally, the cytoplasmic translocation of SUMOylated FoxM1 and its subsequent degradation via ubiquitination were described.^21^ However, DESI1 knockdown did not cause the exclusion of FoxM1 from the nucleus (data not shown).

Interestingly, the enhancement of FoxM1's transcriptional activity has been reported to occur upon its SUMO2 modification.^20^ Notably, SUMOylation of FoxM1b has contrasting effects on the transcription of JNK1 and p21; while it represses p21 genes, it is required for JNK1 transcription.^48^ The introduction of shRNA against DESI1 into cells does not alter the overall cellular pattern of SUMOylation, suggesting that DESI1 substrates are more specific than those of SENP family proteins.^26^ Given this, FoxM1, when deSUMOylated at a specific amino acid residue by DESI1, may elicit effects entirely distinct from those of highly SUMOylated FoxM1 and the SUMOylation‐deficient FoxM1 mutant previously described.^20^, ^21^ In the present study, transfection of FoxM1 with either SUMO1 or SUMO2 enabled the detection of FoxM1 SUMOylation by Western blot analysis, but simultaneous transfection with DESI1 did not significantly alter the band pattern of FoxM1 SUMOylation (data not shown). We hypothesized that DESI1 might deSUMOylate FoxM1 at the specific site among the multiple SUMOylation sites. The overall band pattern of SUMOylated FoxM1 on the Western blot may remain unchanged in this case. The catalytically inactive DESI1 C108S mutant failed to rescue the phenotypes induced by DESI1 knockdown, indicating that catalytic activity of DESI1 is essential for its role in regulating cell division. Identifying the de‐SUMOylation site of FoxM1 by DESI1 is required to conclude the involvement of the de‐SUMOylating activity of DESI1 in regulating FoxM1 transcriptional activity by DESI1.

Aurora B is SUMOylated, a process whose role has been documented.^18^, ^19^ The expression of a SUMO‐deficient Aurora B mutant leads to defects in chromosome segregation and cytokinesis failure.^18^ The Aurora B substrate, CENP‐A, is not phosphorylated at the kinetochore by the SUMO‐deficient Aurora B mutant. Interestingly, the SUMO‐deficient mutation does not decrease, but rather increases, Aurora B kinase activity. Given this, the loss of CENP‐A phosphorylation may be attributed to a defect in Aurora B localization.^18^ In contrast, Aurora B autophosphorylation is enhanced in cells overexpressing the E3 SUMO–protein ligase PIAS3, indicating activation of Aurora B kinase activity by SUMOylation.^19^ This is confirmed by the fact that overexpression of SENP2, a SUMO‐specific isopeptidase, downregulates Aurora B autophosphorylation.^19^ In our current study, DESI1 knockdown accelerated mitotic progression with mis‐segregation of chromosomes, suggesting that an increase in the SUMOylation level of Aurora B could reduce its kinase activity. However, we found that Aurora B protein levels were reduced upon DESI1 knockdown. Although we cannot rule out the involvement of modifications in the SUMOylation levels of Aurora B, the decrease in Aurora B protein levels is primarily responsible for the phenotypes caused by DESI1 knockdown.

Interestingly, we observed a recovery of Aurora B expression levels upon treatment with a proteasome inhibitor, suggesting that DESI1 plays a role in maintaining Aurora B protein stability. How does DESI1 participate in the regulation of Aurora B degradation? SUMOylation contributes to the recruitment of E3 ubiquitin ligases, leading to the degradation of SUMOylated proteins through ubiquitination.^49^, ^50^ SENP6 is a SUMO protease that targets SUMO‐2/3 chains. Depletion of SENP6 results in CENP‐I degradation in a manner dependent on RNF4,^50^ which is a ubiquitin ligase and has four potential SUMO interaction motifs.^49^ Given that Aurora B could be involved in SUMO‐2/3 chain‐mediated recruitment of ubiquitin ligases, DESI1 knockdown may lead to Aurora B degradation via enhancement of SUMO‐2/3 modification. Aurora B degradation is regulated by APC/C‐Cdh1.^51^ Since its subunits, APC2 and APC4, are reportedly regulated for their activity by SUMOylation,^52^ it is plausible that DESI1 knockdown facilitates Aurora B degradation by affecting APC/C activity. Further studies will be required to clarify the mechanism underlying the DESI1 knockdown‐induced Aurora B degradation.

Our findings in the TCGA database indicate that reduced DESI1 expression levels are associated with a poorer prognosis in patients with endometrioid cancer (Figure 7). The chromosome segregation defect caused by DESI1 knockdown (Figure 4 E,F) suggests a role for chromosomal instability in the development of this cancer with low DESI1 expression levels. We found that DESI1 knockdown reduced sensitivity to vincristine, possibly due to attenuation of SAC activity and subsequent induction of mitotic slippage (Figure 8). Cells that prematurely exit cell division in the presence of microtubule‐depolymerizing agents often possess an excess number of centrosomes, leading to abnormal cell division in the subsequent cell cycle. Actually, we observed abnormal cell division, such as multipolar spindle and multipolar cytokinesis (Figure 8E). This suggests that the use of vincristine for cancer cells with decreased levels of DESI1 may increase the risk of cancer development and malignancy via chromosomal instability. Interestingly, the simultaneous use of the APC/C inhibitor with vincristine restored sensitivity to vincristine in DESI1 knockdown cells, presumably by maintaining M‐phase arrest. The combination of microtubule‐targeting agents with the APC/C inhibitor may reduce the dose of microtubule‐targeting agents and their side effects. In conclusion, our study demonstrates that DESI1 is a novel factor regulating cell division by maintaining Aurora B expression levels in an isopeptidase activity‐dependent manner. DESI1 expression levels are associated with the prognosis of patients with cancer. Additionally, DESI1 affects the sensitivity of cancer cells to anticancer agents whose cytotoxicity is dependent on the SAC activity. Future research exploring the regulation of the substrate functions by DESI1 in cell division will be exciting.

## AUTHOR CONTRIBUTIONS

Conceptualization, Yuji Nakayama; Methodology, Yuki Ikeda, Yuji Nakayama; Validation, Yuki Ikeda; Formal Analysis, Yuki Ikeda; Investigation, Yuki Ikeda, Ryuzaburo Yuki, Youhei Saito, Yuji Nakayama; Re‐sources, Yuji Nakayama; Data Curation, Ryuzaburo Yuki, Youhei Saito, Yuji Nakayama; Writing—Original Draft Preparation, Yuki Ikeda, Yuji Nakayama; Writing—Review & Editing, Yuji Nakayama; Visualization, Yuji Nakayama; Supervision, Yuji Nakayama; Project Administration, Yuji Nakayama; Funding acquisition, Yuji Nakayama. All authors were involved in drafting and revising the manuscript.

## DISCLOSURES

The authors declare no conflict of interest.

## Supporting information


Supplementary Figures.

## Data Availability

The data that support the findings of this study are available in the Materials and Methods, Results, and Supplemental information of this article and are available from the corresponding author upon request.
