# Supplementary material for: DeSUMOylating isopeptidase 1 participates in the faithful chromosome segregation and vincristine sensitivity
Source: FASEB J. 2024 Dec 19;38(24):e70261. doi: 10.1096/fj.202401560RR (PMC11656513; doi:10.1096/fj.202401560RR)
Supplement: Supplementary file 1 — Supplementary Figures. [file FSB2-38-e70261-s001.pdf]

Supporting Information

DeSUMOylating isopeptidase 1 participates in the faithful chromosome segregation and vincristine sensitivity

Yuki Ikeda[1], Ryuzaburo Yuki[1], Youhei Saito[1], Yuji Nakayama[1]*

[1]Laboratory of Biochemistry & Molecular Biology, Kyoto Pharmaceutical University, Kyoto 607-8414, Japan

*Corresponding author: Yuji Nakayama, Ph.D.
Laboratory of Biochemistry & Molecular Biology
Kyoto Pharmaceutical University
5 Misasagi-Nakauchi-cho, Yamashina-ku, Kyoto 607-8414, Japan
Phone: +81-75-595-4653; Fax: +81-75-595-4758
Email: nakayama@mb.kyoto-phu.ac.jp

Supplementary Figure 1

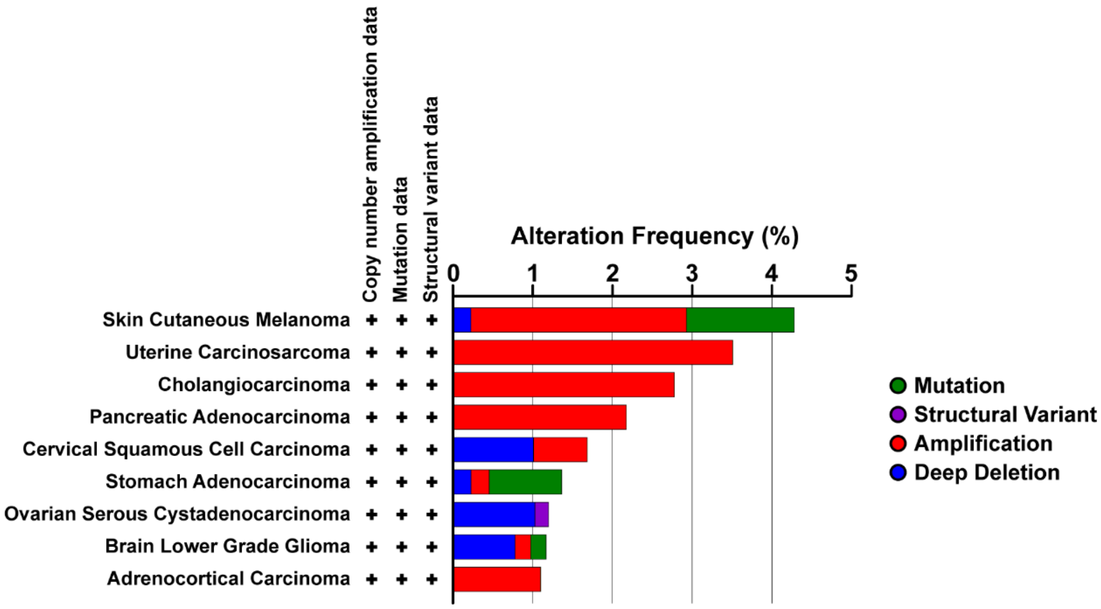

Figure S1. DESI1 is amplified in certain cancer types.
DESI1 gene mutations were extracted for indicated cancer types according to the TCGA database using c-BioPortal (https://www.cbioportal.org).

Supplementary Figure 2

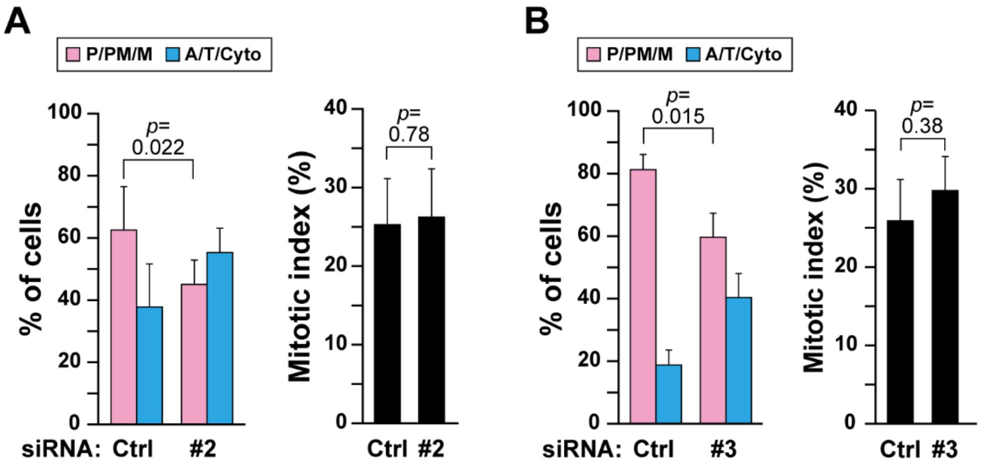

Figure S2. Mitotic progression is expedited by the DESI1 knockdown.
(A, B) HeLa S3 cells underwent treatment with siCtrl, siDESI1 #2, and #3 for a duration of 48 h and were incubated with 6 μM RO-3306 during the final 20 h. Following RO-3306 removal, cells were cultured with flesh medium for 45 min, fixed, and stained for α-tubulin and DNA. M-phase progression was assessed based on the morphologies of DNA and microtubules by categorizing mitotic cells into two groups: from mitotic entry to metaphase (P/PM/M, pink) and after the onset of anaphase (A/T/Cyto, blue). The percentages of each group are displayed in B and C as mean ± SD calculated from six and three independent experiments, respectively (A, n > 220; B, n > 218 per condition). From these experiments, the mitotic index was determined (A, n > 1004; B, n > 1013 per condition). *p*-values were calculated using the Student's *t*-test.

Supplementary Figure 3

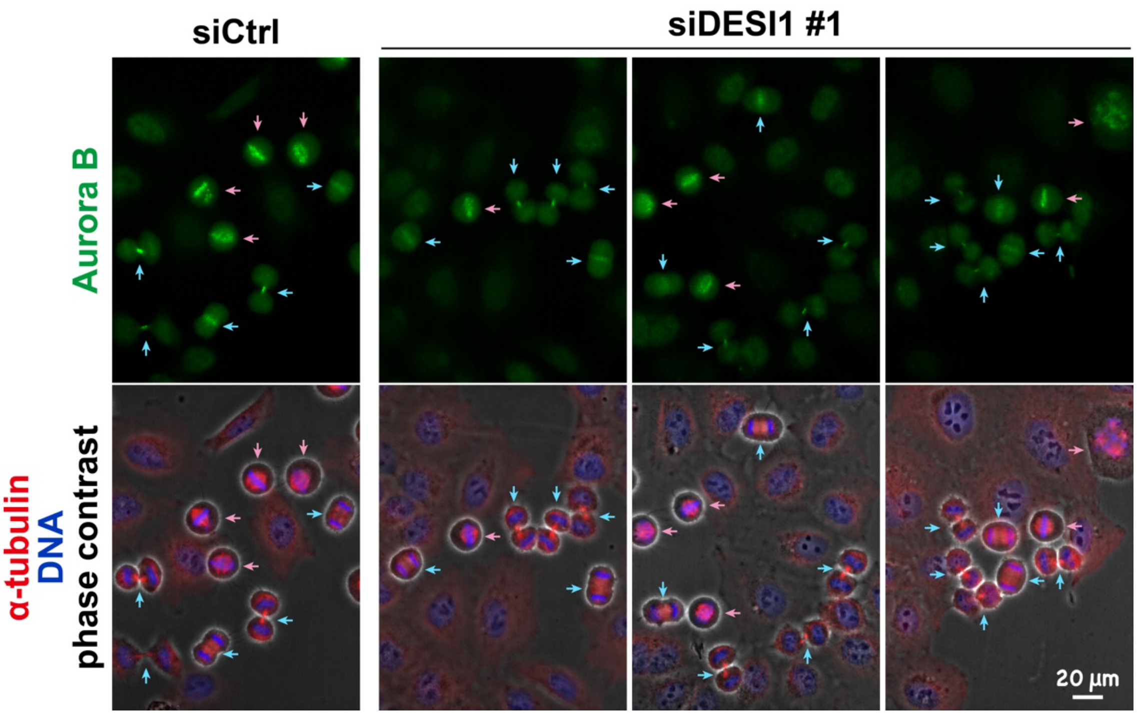

Figure S3. DESI1 knockdown reduces the localization of Aurora B on midzone and midbody. HeLa S3 cells were transfected with siCtrl or siDESI1 #1. After 6 h, the medium was changed with a fresh one and cultured for an additional 22 h. Subsequently, the cells were incubated with 6 µM RO-3306 for 20 h and then cultured for 60 min without RO-3306. The cells were fixed with 4% formaldehyde in PBS (-) and stained for α-tubulin (red), Aurora B (green), and DNA (blue). Scale bar, 20 µm. Pink and pale blue Arrows designate the cells before and after the anaphase onset, respectively.

Supplementary Figure 4

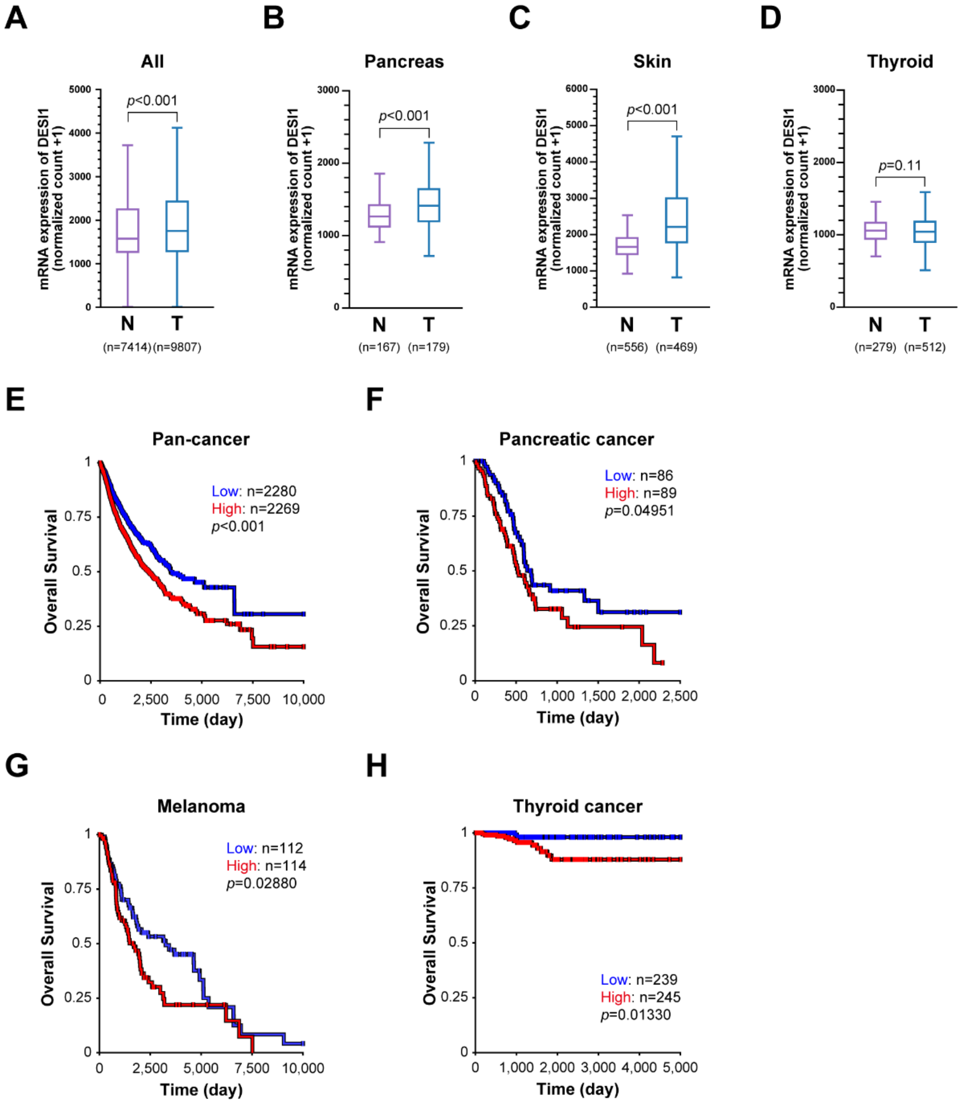

Figure S4. High expression of DESI1 is associated with a poorer prognosis in patients of cancer. (A−D) The relative mRNA expression of DESI1 in normal tissue (N) and Tumor (T) in TCGA datasets were shown. Comparison of mRNA levels between all tissues and tumors (A), normal pancreas and pancreatic adenocarcinoma (B), skin and melanoma (C), and normal thyroid and thyroid cancer (D). *p*-values were calculated by Welch's *t*-test. (E−H) Cancer patients in TCGA datasets were divided into two groups based on DESI1 expression level (E, pan-cancer; F, pancreatic cancer; G, melanoma; H, thyroid cancer), and Kaplan-Meier curves for overall survival are shown. In G and H, the patients were divided according to median DESI1 expression levels. In E and F, the patients in top quartile and bottom quartile in terms of protein expression were compared with respect to overall survival. *p*-values were calculated by log-rank test.

Supplementary Figure 5

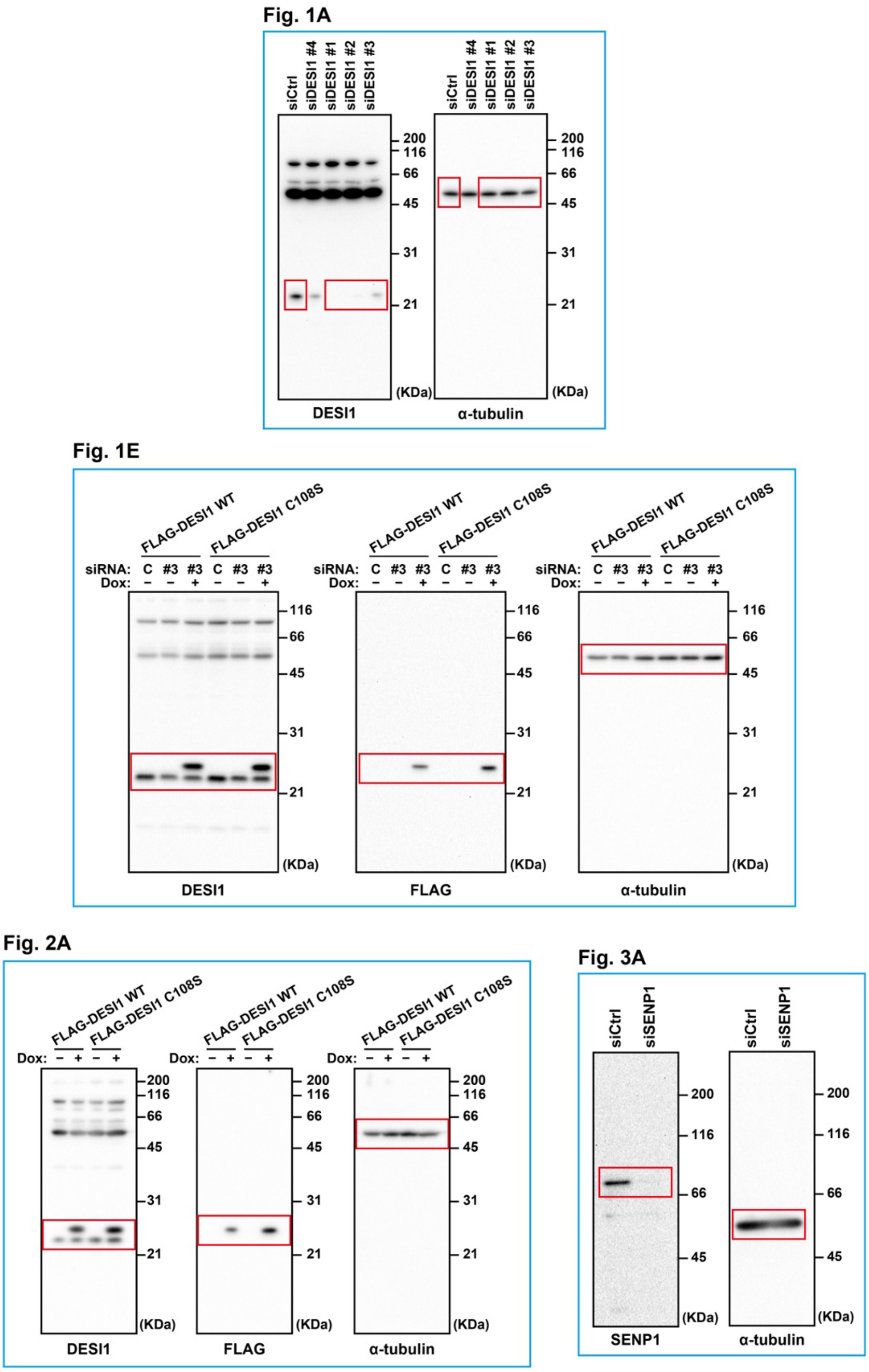

Figure S5. Full-length blots for Figures 1 and 2.

Supplementary Figure 6

Fig. 3E

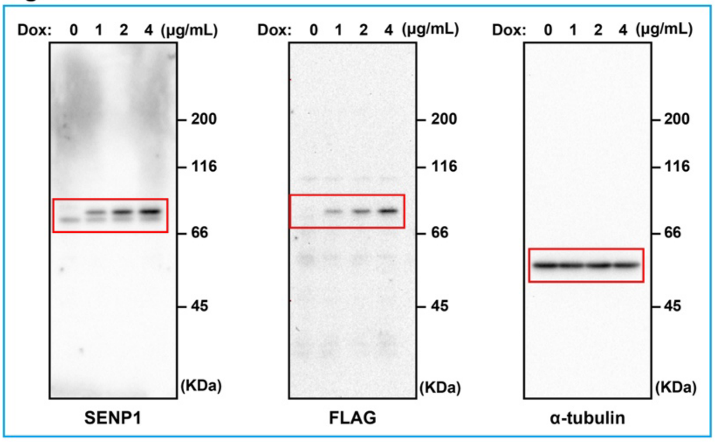

Fig. 5C

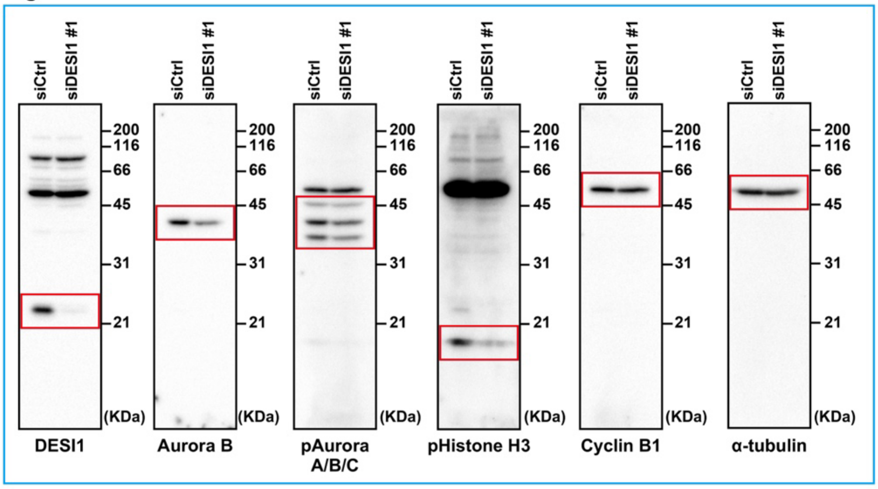

Fig. 5D

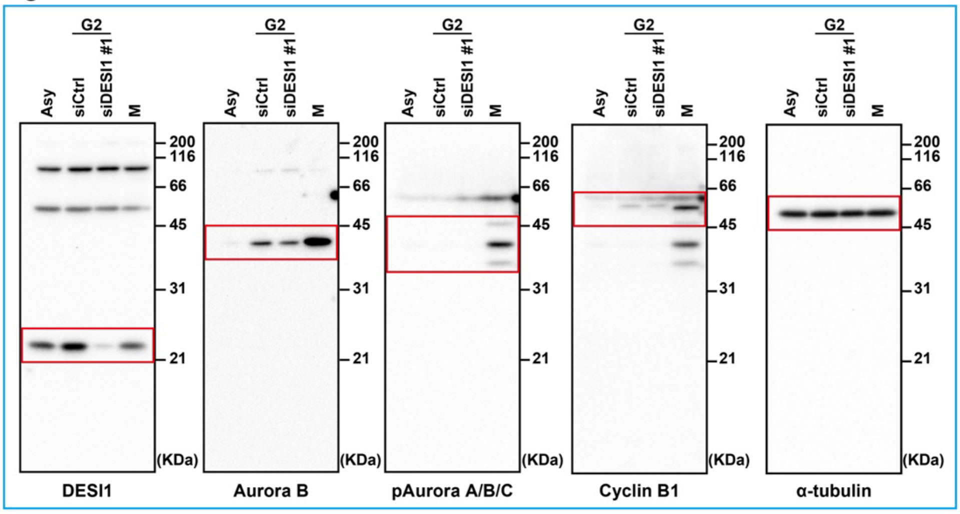

Figure S6. Full-length blots for Figures 3 and 5.

Supplementary Figure 7

Fig. 6B

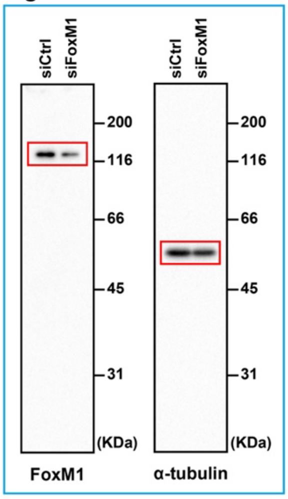

Fig. 6C

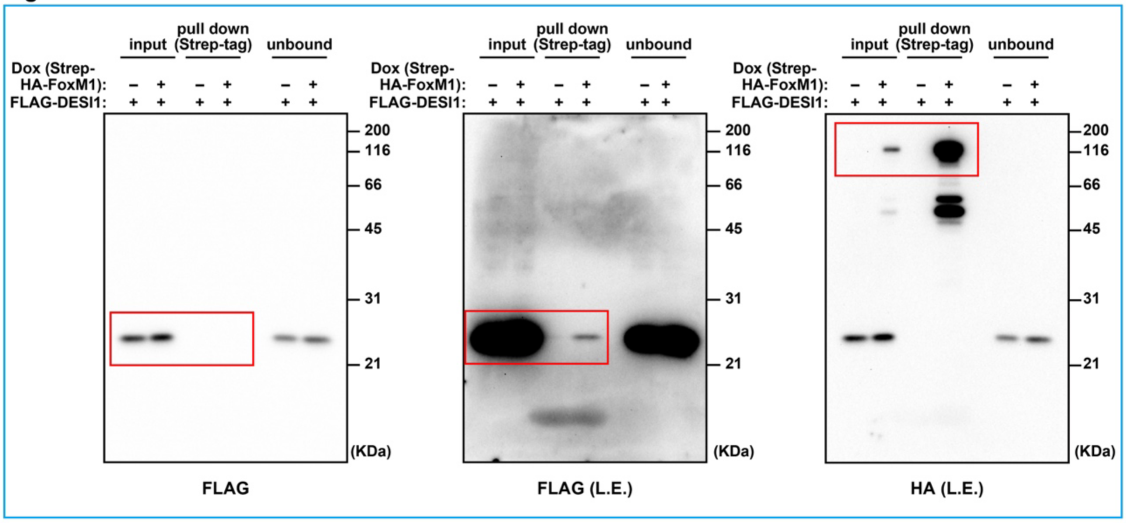

Figure S7. Full-length blots for Figure 6.

Supplementary Figure 8

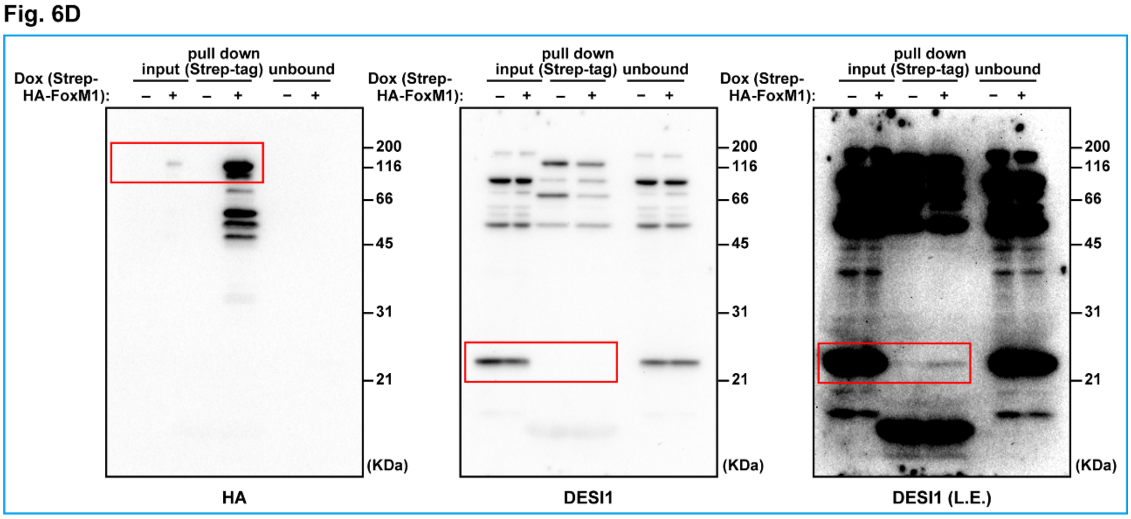

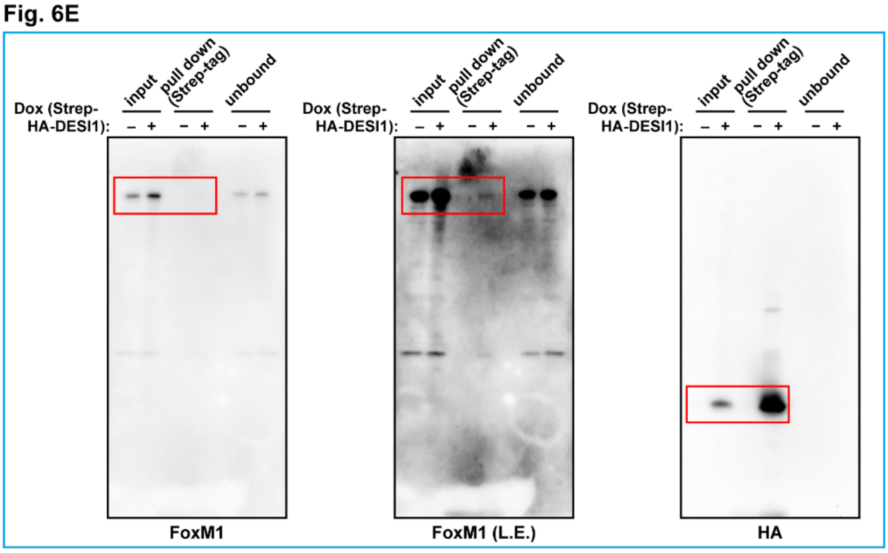

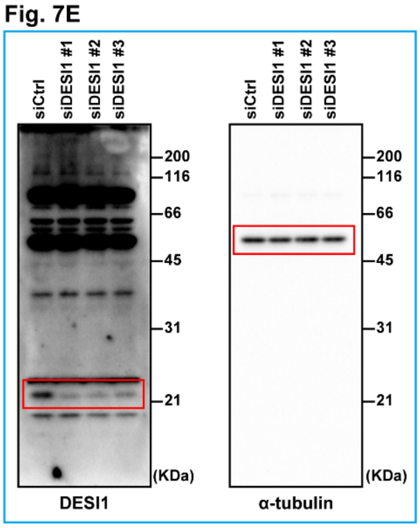

Figure S8. Full-length blots for Figure 6 and 7.
